# Supplementary material for: Determinants of Dutch public health professionals’ intention to use digital contact tracing support tools: A cross-sectional online questionnaire study
Source: PLOS Digit Health. 2024 Feb 14;3(2):e0000425. doi: 10.1371/journal.pdig.0000425 (PMC10866487; doi:10.1371/journal.pdig.0000425)
Supplement: S2 Appendix — (DOCX) [file pdig.0000425.s002.docx]

**S2 Appendix. Questionnaire (translated from Dutch to English)**

***** All questions in this section were asked on 5-point Likert scales

**SECTION 1. Questions about yourself.**

We start with some questions about yourself.

**1.1. What is your age?**

- 18-25
- 26-30
- 31-35
- 36-40
- 41-45
- 46-50
- 51-55
- 56-60
- 61-65
- 65+

**1.2. What is your gender?**

- Male
- Female
- Other, namely: ...

**1.3. In which province are you employed at a PHS?**

- I regularly work elsewhere, for example through a temp agency
- North Holland
- South Holland
- Zeeland
- North Brabant
- Utrecht
- Flevoland
- Friesland
- Groningen
- Drenthe
- Overijssel
- Gelderland
- Limburg

**1.4. What is your primary employment at the PHS?**

- Public health doctor
- Public health doctor (trainee)
- Public health nurse
- Public health nurse (trainee)
- Temporary contact tracer with medical training (in addition to the standard CT-training), such as nursing or medicine
- Temporary contact tracer without medical training (only the CT-training)
- Other, namely: ...

**1.5. How long (approximately) have you been involved with CT for COVID-19?**

- Less than 1 month
- 1-6 months
- 7-12 months
- 1-2 years
- I have no experience with CT for COVID-19

**1.6. Do you have experience with CT for other infectious diseases than COVID-19?**

- Yes
- No

**SECTION 2*. Current COVID-19 CT-practices**

The following statements are about your experiences and opinion regarding the implementation of CT for COVID-19. When responding to the statements, consider how you usually perform CT at the present time.

Complete the sentence below by selecting an answer for [...].

***At the present time, I generally find that performing CT is…***

**2.1. [...]**

- (a lot of work - not much work)

**2.2. [...]**

- (slow - fast)

**2.3. [...]**

- (difficult - easy)

**2.4. [...]**

- (unimportant - important)

**2.5. [...]**

- (unnecessary - necessary)

**2.6. [...]**

- (useless - useful)

**2.7. [...] for stopping the transmission of COVID-19**

- (ineffective - effective)

**2.8. [...] for gaining insight into transmission (spread) of the virus**

- (ineffective - effective)

**SECTION 3*. Involvement of cases and contacts in CT**

The following statements are about your general opinion regarding the involvement of cases and contacts in the implementation of CT for COVID-19.

Fill in [...] for each statement by selecting an answer.

**3.1. I think most people are [...] aware of what CT for COVID-19 entails**

- (not at all - fully)

**3.2. I think most people are [...] aware of the role of CT in combating COVID-19**

- (not at all - fully)

**3.3. Generally, I find the cooperation of cases in CT to be [...]**

- (insufficient - sufficient)

**3.4. Generally, I find the cooperation of contacts in CT to be [...]**

- (insufficient - sufficient)

**3.5. Generally, I find the compliance with advice given during CT to cases (such as testing, isolation, etc.) to be [...]**

- (insufficient - sufficient)

**3.6. Generally, I find the compliance with advice given during CT to contacts (such as testing, quarantine, etc.) to be [...]**

- (insufficient - sufficient)

**3.7. Generally, I think that CT is [...]**

- (entirely the responsibility of the PHS - entirely the responsibility of cases and contacts themselves)

**3.8. Generally, I believe that guidance and control from the PHS are [...] for properly executing CT**

- (not necessary at all - very necessary)

**SECTION 4*. Digitization of CT**

During the COVID-19 pandemic, many digital developments related to CT have emerged or accelerated. These include the development and implementation of new systems such as CoronIT, CoronaMelder, GGDContact, and CoronaCare. The following statements are about your general attitude toward the digitization of CT.

Fill in [...] for each statement by selecting an answer.

**4.1. Digitization makes CT [...] for me**

- (less efficient - more efficient)

**4.2. It takes me [...] time and effort to learn how to use new digital systems for CT**

- (a lot of - not much)

**4.3. I think that digitization of CT is [...] to improve CT now and in the future**

- (unnecessary - necessary)

**4.4. Personal contact with cases is [...] to me in CT**

- (unimportant - important)

**4.5. Personal contact with contacts is [...] to me in CT**

- (unimportant - important)

**4.6. I think the execution of CT can [...] be digitized**

- (not at all - entirely)

**4.7. I have [...] trust in the government/PHS's development of new digital systems for CT**

- (little - a lot of)

**4.8. I have [...] trust in the government/PHS's implementation of new digital systems for CT**

- (little - a lot of)

**4.9. I am [...] concerned about protection and security of (personal) data during the implementation of new digital systems for CT**

- (not very - very)

**4.10. Overall, I have a [...] attitude towards the digitization of CT**

- (negative - positive)

**SECTION 5. Digitally involving cases and contacts in the execution of CT through digital tools**

This section is about different types of digital applications that cases and their contacts may potentially use to support the execution of CT by PHS. The applications focus on three aspects of CT:

**Application 1:** Making an overview of personal data and data about contacts

**Application 2:** Informing contacts

**Application 3:** Monitoring contacts

On the following pages, each type of application is explained in more detail in an image.

Click "Next" to view Application 1! Before answering the questions, make sure to view the image and read the text.

**5.1*. APPLICATION 1**

**Figure 1. Cases collect their personal and their contacts’ data and share these data with PHS using DCTS-tool 1**

**
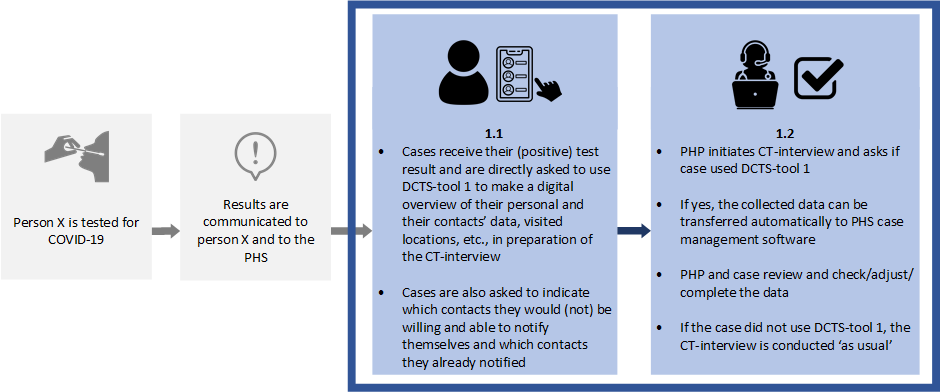
**

For the following statements, we will compare the use of Application 1 in CT for COVID-19 with a situation in which cases do not have the option to digitally collect and provide data to the PHS in preparation of the CT-interview.

Complete each sentence by selecting an answer for [...]. Please consider a situation in CT where no separate guidelines apply, as may be the case in certain settings, contexts, or other exceptional situations (e.g., healthcare facilities).

***If I were able to use Application 1 in CT, I generally think that...***

**5.1.1. I could perform CT [...]**

- (more slowly - more quickly).

**5.1.2. CT would be [...] for me**

- (more work - less work)

**5.1.3. CT would be [...] for me**

- (more difficult - easier)

**5.1.4. Cases would find it [...] to participate in CT**

- (more bothersome - more pleasant)

**5.1.5. The correctness of the collected data in CT would be [...]**

- (worse - better)

**5.1.6. The completeness of the collected data in CT would be [...]**

- (worse - better)

**5.1.7. I would have [...] control over CT**

- (less - more)

**5.1.8. Cases would be [...] willing to participate in CT**

- (insufficiently - sufficiently)

**5.1.9. Cases would have [...] skills to cooperate in CT**

- (insufficient - sufficient)

**5.1.10. I could identify [...] contacts, settings, clusters, etc., in CT**

- (fewer - more)

**5.1.11. I could support cases [...] in CT**

- (worse - better)

**5.1.12. Cases would [...] with the advice given to them**

- (comply worse - comply better)

**5.1.13. If Application 1 were available at my PHS, I would [...] want to use it to collect data from cases and their contacts**

- (definitely not - definitely)

**5.2*. APPLICATION 2**

**Figure 2. Cases digitally inform (a selection of) their contacts themselves using DCTS-tool 2**

**
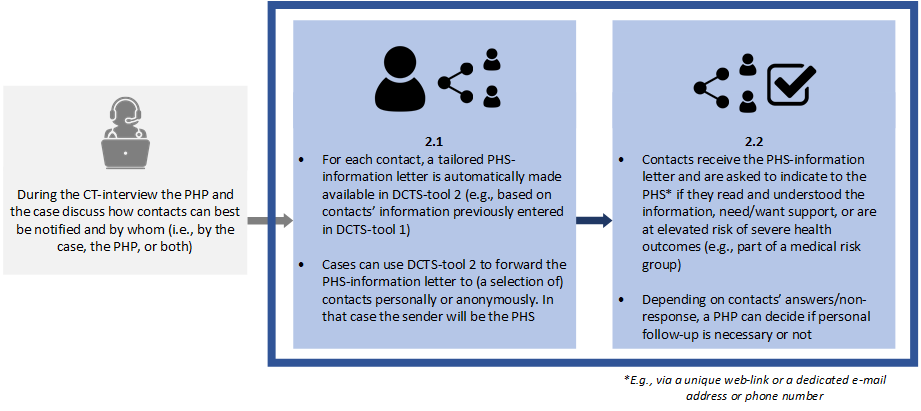
**

For the following statements, we will compare the use of Application 2 in CT for COVID-19 with a situation where informing contacts is only done by the PHS.

Complete each sentence by selecting an answer for [...]. Please consider a situation in CT where no separate guidelines apply, as may be the case in certain settings, contexts, or other exceptional situations (e.g., healthcare facilities).

***If I were able to use Application 2 in CT, I generally think that...***

**5.2.1. I could perform CT [...]**

- (more slowly - more quickly)

**5.2.2. CT would be [...] for me**

- (more work - less work)

**5.2.3. CT would be [...] for me**

- (more difficult - easier)

**5.2.4. Cases would find it [...] to participate in CT**

- (more bothersome - more pleasant)

**5.2.5. Contacts would find it [...] to participate in CT**

- (more bothersome - more pleasant)

**5.2.6. The correctness of the collected data in CT would be [...]**

- (worse - better)

**5.2.7. The completeness of the collected data in CT would be [...]**

- (worse - better)

**5.2.8. I would have [...] control over CT**

- (less - more)

**5.2.9. Cases would be [...] willing to cooperate in CT**

- (insufficiently - sufficiently)

**5.2.10. Cases would have [...] skills to cooperate in CT**

- (insufficient - sufficient)

**5.2.11. I could inform [...] contacts**

- (fewer - more)

**5.2.12. I could inform contacts […]**

- (worse - better)

**5.2.13. I could support cases [...] in CT**

- (worse - better)

**5.2.14. I could support contacts [...] in CT**

- (worse - better)

**5.2.15. Contacts would be [...] to comply with the advice given to them**

- (less likely - more likely)

**5.2.16. If Application 2 were available at my PHS, I would generally [...] want to use it to inform contacts**

- (definitely not - definitely)

**5.3*. APPLICATION 3**

**Figure 3. Contacts self-monitor and digitally register symptoms and health status using DCTS-tool 3**


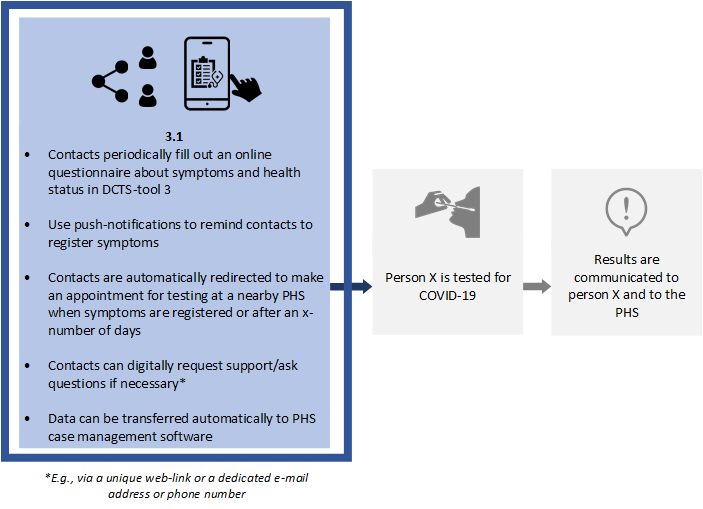


For the following statements, we will compare the use of Application 3 in CT for COVID-19 with a situation where contact monitoring is performed by the PHS.

Complete each sentence by selecting an answer for [...]. Please consider a situation in CT where no separate guidelines apply, as may be the case in certain settings, contexts, or other exceptional situations (e.g., healthcare facilities).

***If I were able to use Application 3 in CT, I generally think that...***

**5.3.1. I could perform CT [...]**

- (more slowly - more quickly)

**5.3.2. CT would be [...] for me**

- (more work - less work)

**5.3.3. CT would be [...] for me**

- (more difficult - easier)

**5.3.4. Contacts would find it [...] to participate in CT**

- (more bothersome - more pleasant)

**5.3.5. The correctness of the collected data in CT would be [...]**

- (worse - better)

**5.3.6. The completeness of the collected data in CT would be [...]**

- (worse - better)

**5.3.7. I would have [...] control over CT**

- (less - more)

**5.3.8. Contacts would be [...] willing to participate in CT**

- (insufficiently - sufficiently)

**5.3.9. Contacts would have [...] skills to cooperate in CT**

- (insufficient - sufficient)

**5.3.10. I could support contacts [...] in CT**

- (worse - better)

**5.3.11. Contacts would [...] with the advice given to them**

- (comply worse - comply better)

**5.3.12. If Application 3 were available at my PHS, I would generally [...] want to use it for monitoring contacts**

- (definitely not - definitely)

**SECTION 6. Conclusion**

6.1. Do you have any comments about the questionnaire that you would like to share?

- (open field)

6.2. Do you have any suggestions regarding the digitization of CT that you would like to share?

- (open field)

**THANK YOU FOR YOUR PARTICIPATION.**
