# Supplementary material for: Determinants of Dutch public health professionals’ intention to use digital contact tracing support tools: A cross-sectional online questionnaire study
Source: PLOS Digit Health. 2024 Feb 14;3(2):e0000425. doi: 10.1371/journal.pdig.0000425 (PMC10866487; doi:10.1371/journal.pdig.0000425)

**S3 Appendix. Supplementary statistical analyses**

**Table A. PHPs’ intention to use DCTS-tools 1, 2, and 3.**

| **Questionnaire item** | **Individuals who completed the online questionnaire (N=641)** | |
| --- | --- | --- |
| **Intention to use DCTS-tool 1** (%) | |  |
| - Definitely | | 147 (20.9%) |
| - Probably | | 306 (43.6%) |
| - Maybe | | 168 (23.9%) |
| - Probably not | | 64 (9.1%) |
| - Definitely not | | 17 (2.4%) |
| **Intention to use DCTS-tool 2** (%) | |  |
| - Definitely | | 96 (14.5%) |
| - Probably | | 287 (43.5%) |
| - Maybe | | 184 (27.9%) |
| - Probably not | | 75 (11.4%) |
| - Definitely not | | 18 (2.7%) |
| **Intention to use DCTS-tool 3** (%) | |  |
| - Definitely | | 121 (18.9%) |
| - Probably | | 233 (36.3%) |
| - Maybe | | 164 (25.6%) |
| - Probably not | | 98 (15.3%) |
| - Definitely not | | 25 (3.9%) |

**Table B. Bivariate associations between predictors and PHPs’ intention to use DCTS-tools 1/2/3 in CT for COVID-19.** In our bivariate analyses, we treated ‘intention’ as a variable with an interval/ordinal measurement scale with values ranging from 0 (very negative/definitely not) to 4 (very positive/definitely). Bivariate associations were computed using spearman correlations (for interval/ordinal independent variables) and the Wilcoxon rank-sum test or the Kruskal-Wallis test (for categorical independent variables with 2 or more levels, respectively).

| **QUESTIONNAIRE SECTION 1: SOCIO-DEMOGRAPHIC AND PROFESSIONAL CHARACTERISTICS** | | | | | |
| --- | --- | --- | --- | --- | --- |
| Questionnaire item | Individuals who completed the online questionnaire (N= 641) | Bivariate association with intention to use DCTS-tool 1 | Bivariate association with intention to use DCTS-tool 2 | Bivariate association with intention to use DCTS-tool 3 |  |
| **1.1. Age** (Mean (SD); Med (IQR)) | Mean (SD) = 40.7 (15.3)  Med (IQR) = 38 (28-53) | S = 38329947  p-value = <0.01**  rho = 0.130 | S = 38971870  p-value = 0.01**  rho = 0.112 | S = 41803188  p-value = 0.23  rho = 0.048 |  |
| **1.2. Gender** (%) |  | W = 39165  p-value = 0.38  r = 0.035 | W = 39812  p-value = 0.59  r = 0.022 | W = 42323  p-value = 0.47  r = 0.028 |  |
| - Female | 462 (72.1%) |  |  |  |  |
| - Male | 177 (27.6%) |  |  |  |  |
| - Non-binary*** | 2 (0.3%) |  |  |  |  |
| **1.3. Province of employment** (%) |  | Kruskal-Wallis X^2^ = 11.22  df = 10  p-value = 0.34  eta^2^ = 0.002 | Kruskal-Wallis X^2^= 6.74  df = 10  p-value = 0.75  eta^2^ = -0.005 | Kruskal-Wallis X^2^ = 16.13  df = 10  p-value = 0.1  eta^2^ = 0.01 |  |
| - Drenthe *** | 4 (0.6%) |  |  |  |  |
| - Flevoland | 15 (2.3%) |  |  |  |  |
| - Friesland | 71 (11.1%) |  |  |  |  |
| - Gelderland | 143 (22.3%) |  |  |  |  |
| - Groningen | 27 (4.2%) |  |  |  |  |
| - Limburg | 58 (9.0%) |  |  |  |  |
| - Noord-Brabant | 37 (5.8%) |  |  |  |  |
| - Noord-Holland | 70 (10.9%) |  |  |  |  |
| - Overijssel | 70 (10.9%) |  |  |  |  |
| - Utrecht | 57 (8.9%) |  |  |  |  |
| - Zeeland | 34 (5.3%) |  |  |  |  |
| - Zuid-Holland | 50 (7.8%) |  |  |  |  |
| - Multiple *** | 5 (0.8%) |  |  |  |  |
| **1.4. Primary employment at PHS** (%) |  | Kruskal-Wallis X^2^ = 14.65  df = 3  p-value = <0.01**  eta^2^ = 0.018 | Kruskal-Wallis X^2^ = 3.77  df = 3  p-value = 0.29  eta^2^ = 0.001 | Kruskal-Wallis X^2^= 3.41  df = 3  p-value = 0.33  eta^2^ = <0.001 |  |
| - CT-staff (manager/coordinator) | 25 (3.9%) |  |  |  |  |
| - CT-staff (executive) | 538 (83.9%) |  |  |  |  |
| - PHS-doctor | 39 (6.1%) |  |  |  |  |
| - PHS-nurse | 32 (5.0%) |  |  |  |  |
| - Other (conversation coach, health educator, policy advisor) *** | 7 (1.1%) |  |  |  |  |
| **1.5. Experience with CT for COVID-19 (time)** (%) |  | S = 49233811  p-value = <0.01**  rho = -0.122 | S = 43820345  p-value = 0.97  rho = 0.002 | S = 45474418  p-value = 0.36  rho = -0.036 |  |
| - No specific COVID-19 CT experience | 15 (2.3%) |  |  |  |  |
| - <1 month | 13 (2.0%) |  |  |  |  |
| - 1-6 months | 137 (21.4%) |  |  |  |  |
| - 7-12 months | 92 (14.4%) |  |  |  |  |
| - 1-2 years | 384 (59.9%) |  |  |  |  |
| **1.6. Experience with CT in general** (%) |  | W = 17989  p-value = 0.048  r = 0.078 | W = 18654  p-value = 0.012*  r = 0.01 | W = 16537  p-value = 0.44  r = 0.03 |  |
| - No | 588 (91.7%) |  |  |  |  |
| - Yes | 53 (8.3%) |  |  |  |  |
| **QUESTIONNAIRE SECTION 2: BELIEFS REGARDING CURRENT CT PRACTICES** | | | | | |
| Questionnaire item | Individuals who completed the online questionnaire (N= 641) | Bivariate association with intention to use DCTS-tool 1 | Bivariate association with intention to use DCTS-tool 2 | Bivariate association with intention to use DCTS-tool 3 |  |
| **2.1. Current perceived workload of CT for PHPs (on average, for conducting one case investigation)** (Mean (SD); Med (IQR))   - Very much work [0] – [4] very little work [4] (5-point Likert scale) | Mean (SD) = 1.97 (0.89)  Med (IQR) = 2 (1-3) | S = 46879093  p-value = 0.086  rho = -0.068 | S = 43404653  p-value = 0.78  rho = 0.011 | S = 42324689  p-value = 0.37  rho = 0.036 |  |
| **2.2. Current perceived speed of CT (on average, for conducting one case investigation)** (Mean (SD); Med (IQR))   - Very slow [0] – very fast [4] (5-point Likert scale) | Mean (SD) = 2.16 (0.91)  Med (IQR) = 2 (2-3) | S = 47538047  p-value = 0.04*  rho = -0.083 | S = 46043463  p-value = 0.22  rho = -0.049 | S = 43645407  p-value = 0.89  rho = 0.006 |  |
| **2.3. Current perceived difficulty of CT (on average, for conducting one case investigation)** (Mean (SD); Med (IQR))   - Very difficult [0] – very easy [4] (5-point Likert scale) | Mean (SD) = 2.7 (0.77)  Med (IQR) = 3 (2-3) | S = 43899027  p-value = 1  rho = 0 | S = 42995383  p-value = 0.6  rho = 0.021 | S = 42321287  p-value = 0.36  rho = 0.036 |  |
| **2.4. Current perceived importance of CT** (Mean (SD); Med (IQR))   - Very unimportant [0] – very important [4] (5-point Likert scale) | Mean (SD) = 2.39 (0.94)  Med (IQR) = 3 (2-3) | S = 44904172  p-value = 0.56  rho = -0.023 | S = 43550921  p-value = 0.84  rho = 0.008 | S = 46035407  p-value = 0.22  rho = -0.049 |  |
| **2.5. Current perceived necessity of CT** (Mean (SD); Med (IQR))   - Very unnecessary [0] – very necessary [4] (5-point Likert scale) | Mean (SD) = 2.4 (0.97)  Med (IQR) = 3 (2-3) | S = 46076217  p-value = 0.21  rho = -0.05 | S = 44732308  p-value = 0.63  rho = -0.019 | S = 45768045  p-value = 0.28  rho = -0.043 |  |
| **2.6. Current perceived usefulness of CT** (Mean (SD); Med (IQR))   - Very useless [0] – very useful [4] (5-point Likert scale) | Mean (SD) = 2.38 (0.97)  Med (IQR) = 3 (2-3) | S = 44426293  p-value = 0.76  rho = -0.012 | S = 42527553  p-value = 0.43  rho = 0.031 | S = 46177035  p-value = 0.19  rho = -0.052 |  |
| **2.7. Current perceived effectiveness of CT for stopping transmission of COVID-19** (Mean (SD); Med (IQR))   - Very ineffective [0] – very effective [4] (5-point Likert scale) | Mean (SD) = 1.94 (1.1)  Med (IQR) = 2 (1-3) | S = 47035968  p-value = 0.07  rho = -0.072 | S = 44088439  p-value = 0.91  rho = -0.004 | S = 42884597  p-value = 0.56  rho = 0.023 |  |
| **2.8. Current perceived effectiveness of CT for gaining insight into transmission of COVID-19** (Mean (SD); Med (IQR))   - Very ineffective [0] – very effective [4] (5-point Likert scale) | Mean (SD) = 2.47 (1.14)  Med (IQR) = 3 (2-3) | S = 46089149  p-value = 0.22  rho = -0.05 | S = 43815225  p-value = 0.96  rho = 0.002 | S = 43972571  p-value = 0.96  rho = -0.002 |  |
| **QUESTIONNAIRE SECTION 3: BELIEFS REGARDING ROLES AND RESPONSIBILITIES OF CASES, CONTACTS, AND PHS IN CT FOR COVID-19** | | | | | |
| Questionnaire item | Individuals who completed the online questionnaire (N= 641) | Bivariate association with intention to use DCTS-tool 1 | Bivariate association with intention to use DCTS-tool 2 | Bivariate association with intention to use DCTS-tool 3 |  |
| **3.1. Belief that cases/contacts know wat CT is and what it entails** (Mean (SD); Med (IQR))   - Completely do not know [0] - completely do know [4] (5-point Likert scale) | Mean (SD) = 2.23 (0.94)  Med (IQR) = 3 (1-3) | S = 40768316  p-value = 0.07  rho = 0.071 | S = 38661549  p-value = <0.01**  rho = 0.119 | S = 42331035  p-value = 0.37  rho = 0.036 |  |
| **3.2. Belief that cases/contacts are aware of the role of CT in combating COVID-19** (Mean (SD); Med (IQR))   - Completely unaware [0] - completely aware [4] (5-point Likert scale) | Mean (SD) = 1.78 (0.96)  Med (IQR) = 2 (1-3) | S = 39576030  p-value = 0.01*  rho = 0.098 | S = 40252517  p-value = 0.036  rho = 0.083 | S = 42168317  p-value = 0.32  rho = 0.039 |  |
| **3.3. Perceived cooperation of cases in CT** (Mean (SD); Med (IQR))   - Insufficient [0] – sufficient [4] (5-point Likert scale) | Mean (SD) = 3.02 (0.68)  Med (IQR) = 3 (3-3) | S = 40765606  p-value = 0.07  rho = 0.071 | S = 42254852  p-value = 0.34  rho = 0.038 | S = 42247394  p-value = 0.34  rho = 0.038 |  |
| **3.4. Perceived cooperation of contacts in CT** (Mean (SD); Med (IQR))   - Insufficient [0] – sufficient [4] (5-point Likert scale) | Mean (SD) = 2.41 (0.87)  Med (IQR) = 3 (2-3) | S = 43639563  p-value = 0.88  rho = 0.006 | S = 42502308  p-value = 0.42  rho = 0.032 | S = 43898186  p-value = 1  rho = 0 |  |
| **3.5. Perceived compliance with CT-measures and guidelines of cases** (Mean (SD); Med (IQR))   - Insufficient [0] – sufficient [4] (5-point Likert scale) | Mean (SD) = 2.69 (0.73)  Med (IQR) = 3 (2-3) | S = 42467892  p-value = 0.41  rho = 0.033 | S = 42820809  p-value = 0.54  rho = 0.024 | S = 44031946  p-value = 0.94  rho = -0.003 |  |
| **3.6. Perceived compliance with CT-measures and guidelines of contacts** (Mean (SD); Med (IQR))   - Insufficient [0] – sufficient [4] (5-point Likert scale) | Mean (SD) = 2.38 (0.79)  Med (IQR) = 2 (2-3) | S = 44464434  p-value = 0.74  rho = -0.013 | S = 43314113  p-value = 0.74  rho = 0.013 | S = 45437549  p-value = 0.37  rho = -0.035 |  |
| **3.7. Belief that CT mainly is the responsibility of PHS or cases/contacts** (Mean (SD); Med (IQR))   - Completely of PHS [0] – completely of cases and contacts [4] (5-point Likert scale) | Mean (SD) = 1.72 (0.75)  Med (IQR) = 2 (1-2) | S = 39789868  p-value = 0.02*  rho = 0.094 | S = 39766586  p-value = 0.02*  rho = 0.094 | S = 39465246  p-value = 0.01*  rho = 0.101 |  |
| **3.8. Perceived need for PHS control to ensure adequate execution of CT** (Mean (SD); Med (IQR))   - Very unnecessary [0] – very necessary [4] (5-point Likert scale) | Mean (SD) = 2.94 (0.79)  Med (IQR) = 3 (3-3) | S = 47286414  p-value = 0.05*  rho = -0.077 | S = 48121161  p-value = 0.01*  rho = -0.0962618820642724 | S = 46171365  p-value = 0.19  rho = -0.052 |  |
| QUESTIONNAIRE SECTION 4: BELIEFS REGARDING DIGITALIZATION OF CT | | | | | |
| Questionnaire item | Individuals who completed the online questionnaire (N= 641) | Bivariate association with intention to use DCTS-tool 1 | Bivariate association with intention to use DCTS-tool 2 | Bivariate association with intention to use DCTS-tool 3 |  |
| **4.1.** **Anticipated effect of digitalization on the efficiency of CT** (Mean (SD); Med (IQR))   - Much more efficient [0] – much less efficient [4] (5-point Likert scale) | Mean (SD) = 2.92 (0.84)  Med (IQR) = 3 (3-3) | S = 31520200  p-value = <0.01**  rho = 0.282 | S = 37509799  p-value = <0.01**  rho = 0.145 | S = 35753133  p-value = <0.01*  rho = 0.185 |  |
| **4.2. Anticipated effort required to learn using new digital systems for CT** (Mean (SD); Med (IQR))   - Very much effort [0] – very little effort [4] (5-point Likert scale) | Mean (SD) = 2.94 (0.84)  Med (IQR) = 3 (2-4) | S = 38127851  p-value = <0.01**  rho = 0.131 | S = 40142001  p-value = 0.03*  rho = 0.086 | S = 38588866  p-value = <0.01*  rho = 0.121 |  |
| **4.3.** **Perceived need for digitalization to improve CT** (Mean (SD); Med (IQR))   - Very unnecessary [0] – very necessary [4] (5-point Likert scale) | Mean (SD) = 3.05 (0.77)  Med (IQR) = 3 (3-4) | S = 31005330  p-value = <0.01**  rho = 0.294 | S = 34416771  p-value = <0.01**  rho = 0.216 | S = 35320302  p-value = <0.01**  rho = 0.195 |  |
| **4.4.** **Perceived importance of personal contact with cases in CT** (Mean (SD); Med (IQR))   - Very unimportant [0] – very important [4] (5-point Likert scale) | Mean (SD) = 3.23 (0.77)  Med (IQR) = 3 (3-4) | S = 50117046  p-value = <0.01**  rho = -0.142 | S = 47507678  p-value = 0.04*  rho = -0.082 | S = 46558603  p-value = 0.12  rho = -0.061 |  |
| **4.5.** **Perceived importance of personal contact with contacts in CT** (Mean (SD); Med (IQR))   - Very unimportant [0] – very important [4] (5-point Likert scale) | Mean (SD) = 2.35 (1.01)  Med (IQR) = 2 (2-3) | S = 47606571  p-value = 0.03*  rho = -0.085 | S = 47839156  p-value = 0.02*  rho = -0.09 | S = 465216067  p-value = 0.13  rho = -0.06 |  |
| **4.6.** **Anticipated extent to which CT can be digitalized** (Mean (SD); Med (IQR))   - Cannot be digitalized at all [0] – can be fully digitalized [4] (5-point Likert scale) | Mean (SD) = 2.45 (0.91)  Med (IQR) = 3 (2-3) | S = 32523148  p-value = <0.01**  rho = 0.259 | S = 35141847  p-value = <0.01**  rho = 0.199 | S = 35829665  p-value = <0.01**  rho = 0.184 |  |
| **4.7.** **Trust in development of novel digital systems for CT by government/PHS** (Mean (SD); Med (IQR))   - Very little trust [0] – very much trust [4] (5-point Likert scale) | Mean (SD) = 2.1 (0.93)  Med (IQR) = 2 (2-3) | S = 36617793  p-value = <0.01**  rho = 0.166 | S = 35720434  p-value = <0.01**  rho = 0.186 | S = 34649842  p-value = <0.01**  rho = 0.211 |  |
| **4.8.** **Trust in implementation of novel digital systems for CT by government/PHS** (Mean (SD); Med (IQR))   - Very little trust [0] – very much trust [4] (5-point Likert scale) | Mean (SD) = 2.05 (0.91)  Med (IQR) = 2 (1-3) | S = 37412409  p-value = <0.01**  rho = 0.148 | S = 37364119  p-value = <0.01**  rho = 0.149 | S = 36996449  p-value = <0.01**  rho = 0.157 |  |
| **4.9.** **Worry about data security and privacy protection in the context of digitalization of CT** (Mean (SD); Med (IQR))   - Not worried at all [0] – Very worried [4] (5-point Likert scale) | Mean (SD) = 1.88 (0.98)  Med (IQR) = 2 (1-3) | S = 48187251  p-value = 0.01*  rho = -0.098 | S = 44993196  p-value = 0.53  rho = -0.025 | S = 46652610  p-value = 0.11  rho = -0.063 |  |
| **4.10.** **Attitude towards digitalization of CT** (Mean (SD); Med (IQR))   - Very negative [0] – Very positive [4] (5-point Likert scale) | Mean (SD) = 2.75 (0.77)  Med (IQR) = 3 (2-3) | S = 27571819  p-value = <0.01**  rho = 0.372 | S = 30885305  p-value = <0.01**  rho = 0.296 | S = 30529236  p-value = <0.01**  rho = 0.305 |  |
| QUESTIONNAIRE SECTION 5: BELIEFS REGARDING BENEFITS AND CHALLENGES OF DCTS-TOOL 1 | | | | | |
| Questionnaire item | Individuals who completed the online questionnaire (N= 641) | Bivariate association with intention to use DCTS-tool 1 | Bivariate association with intention to use DCTS-tool 2 (N/A) | Bivariate association with intention to use DCTS-tool 3 (N/A) |  |
| **5.1.1.** **Anticipated effect on speed of CT** (Mean (SD); Med (IQR))   - Much slower [0] – much faster [4] (5-point Likert scale) | Mean (SD) = 2.75 (0.78)  Med (IQR) = 3 (2-3) | S = 20930692  p-value = <0.01**  rho = 0.523 | N/A | N/A |  |
| **5.1.2.** **Anticipated effect on the workload of PHPs in CT** (Mean (SD); Med (IQR))   - Much more work [0] – much less work [4] (5-point Likert scale) | Mean (SD) = 2.76 (0.82)  Med (IQR) = 3 (2-3) | S = 23109863  p-value = <0.01**  rho = 0.474 | N/A | N/A |  |
| **5.1.3.** **Anticipated effect on the difficulty of CT** (Mean (SD); Med (IQR))   - Much more difficult [0] – much easier [4] (5-point Likert scale) | Mean (SD) = 2.65 (0.75)  Med (IQR) = 3 (2-3) | S = 23779229  p-value = <0.01**  rho = 0.458 | N/A | N/A |  |
| **5.1.4.** **Anticipated effect on the pleasantness to participate in CT for cases** (Mean (SD); Med (IQR))   - Much less pleasant [0] – much more pleasant [4] (5-point Likert scale) | Mean (SD) = 1.94 (0.92)  Med (IQR) = 2 (1-3) | S = 24806576  p-value = <0.01**  rho = 0.435 | N/A | N/A |  |
| **Anticipated effect on the pleasantness to participate in CT for contacts** (Mean (SD); Med (IQR))   - Much less pleasant [0] – much more pleasant [4] (5-point Likert scale) | N/A | N/A | NA | NA |  |
| **5.1.5.** **Anticipated effect on the correctness of data collected in CT (Mean (SD); Med (IQR))**   - Much less correct [0] – much more correct [4] (5-point Likert scale) | Mean (SD) = 1.98 (0.96)  Med (IQR) = 2 (1-3) | S = 24980076  p-value = <0.01**  rho = 0.431 | NA | N/A |  |
| **5.1.6.** **Anticipated effect on the completeness of data collected in CT (Mean (SD); Med (IQR))**   - Much less complete [0] – much more complete [4] (5-point Likert scale) | Mean (SD) = 2 (0.995)  Med (IQR) = 2 (1-3) | S = 25275840  p-value = <0.01**  rho = 0.424 | N/A | N/A |  |
| **5.1.7.** **Anticipated effect on the control of PHPs over the CT-process (Mean (SD); Med (IQR))**   - Much less control [0] – much more control [4] (5-point Likert scale) | Mean (SD) = 1.71 (0.95)  Med (IQR) = 2 (1-2) | S = 23346546  p-value = <0.01**  rho = 0.468 | N/A | N/A |  |
| **5.1.8.** **Anticipated willingness of cases to participate in CT with DCTS-tool (Mean (SD); Med (IQR))**   - Insufficiently willing [0] – sufficiently willing [4] (5-point Likert scale) | Mean (SD) = 1.95 (0.94)  Med (IQR) = 2 (1-3) | S = 24692846  p-value = <0.01**  rho = 0.437 | N/A | N/A |  |
| **Anticipated willingness of contacts to participate in CT with DCTS-tool (Mean (SD); Med (IQR))**   - Insufficiently willing [0] – sufficiently willing [4] (5-point Likert scale) | N/A | N/A | N/A | N/A |  |
| **5.1.9.** **Anticipated skills of cases to participate in CT with DCTS-tool (Mean (SD); Med (IQR))**   - Insufficiently skilled [0] – sufficiently skilled [4] (5-point Likert scale) | Mean (SD) = 2.1 (0.83)  Med (IQR) = 2 (2-3) | S = 25162114  p-value = <0.01**  rho = 0.427 | N/A | N/A |  |
| **Anticipated skills of contacts to participate in CT with DCTS-tool (Mean (SD); Med (IQR))**   - Insufficiently skilled [0] – sufficiently skilled [4] (5-point Likert scale) | N/A | N/A | N/A | N/A |  |
| **5.1.10.** **Anticipated effect on the number of identified cases, settings, and clusters in CT (Mean (SD); Med (IQR))**   - Much less [0] – much more [4] (5-point Likert scale) | Mean (SD) = 2.28 (0.94)  Med (IQR) = 2 (2-3) | S = 27832529  p-value = <0.01**  rho = 0.366 | N/A | N/A |  |
| **Anticipated effect on the number of contacts reached in CT (Mean (SD); Med (IQR))**   - Much less [0] – much more [4] (5-point Likert scale) | N/A | N/A | N/A | N/A |  |
| **Anticipated effect on adequate notification/informing of contacts (Mean (SD); Med (IQR))**   - Much worse [0] – much better [4] (5-point Likert scale) | N/A | N/A | N/A | N/A |  |
| **5.1.11.** **Anticipated effect on support that PHPs can offer to cases (Mean (SD); Med (IQR))**   - Much worse [0] – much better [4] (5-point Likert scale) | Mean (SD) = 2.35 (0.82)  Med (IQR) = 2 (2-3) | S = 23185512  p-value = <0.01**  rho = 0.472 | N/A | N/A |  |
| **Anticipated effect on support that PHPs can offer to contacts (Mean (SD); Med (IQR))**   - Much worse [0] – much better [4] (5-point Likert scale) | N/A | N/A | N/A | N/A |  |
| **5.1.12.** **Anticipated effect on compliance of cases with CT-measures and guidelines (Mean (SD); Med (IQR))**   - Much worse [0] – much better [4] (5-point Likert scale) | Mean (SD) = 1.89 (0.73)  Med (IQR) = 2 (2-2) | S = 27691422  p-value = <0.01**  rho = 0.369 | N/A | N/A |  |
| **Anticipated effect on compliance of contacts with CT-measures and guidelines (Mean (SD); Med (IQR))**   - Much worse [0] – much better [4] (5-point Likert scale) | N/A | N/A | N/A | N/A |  |
| QUESTIONNAIRE SECTION 5: BELIEFS REGARDING BENEFITS AND CHALLENGES OF DCTS-TOOL 2 | | | | | |
| Questionnaire item | Individuals who completed the online questionnaire (N= 641) | Bivariate association with intention to use DCTS-tool 1 (N/A) | Bivariate association with intention to use DCTS-tool 2 | Bivariate association with intention to use DCTS-tool 3 (N/A) |  |
| **5.2.1. Anticipated effect on speed of CT** (Mean (SD); Med (IQR))   - Much slower [0] – much faster [4] (5-point Likert scale) | Mean (SD) = 2.69 (0.79)  Med (IQR) = 3 (2-3) | N/A | S = 24865528  p-value = <0.01**  rho = 0.434 | N/A |  |
| **5.2.2. Anticipated effect on the workload of PHPs in CT** (Mean (SD); Med (IQR))   - Much more work [0] – much less work [4] (5-point Likert scale) | Mean (SD) = 2.75 (0.88)  Med (IQR) = 3 (2-3) | N/A | S = 29604446  p-value = <0.01**  rho = 0.326 | N/A |  |
| **5.2.3. Anticipated effect on the difficulty of CT** (Mean (SD); Med (IQR))   - Much more difficult [0] – much easier [4] (5-point Likert scale) | Mean (SD) = 2.59 (0.74)  Med (IQR) = 3 (2-3) | N/A | S = 25572751  p-value = <0.01**  rho = 0.417 | N/A |  |
| **5.2.4. Anticipated effect on the pleasantness to participate in CT for cases** (Mean (SD); Med (IQR))   - Much less pleasant [0] – much more pleasant [4] (5-point Likert scale) | Mean (SD) = 2.12 (0.83)  Med (IQR) = 2 (2-3) | N/A | S = 23533028  p-value = <0.01**  rho = 0.464 | N/A |  |
| **5.2.5. Anticipated effect on the pleasantness to participate in CT for contacts** (Mean (SD); Med (IQR))   - Much less pleasant [0] – much more pleasant [4] (5-point Likert scale) | Mean (SD) = 2.16 (0.87)  Med (IQR) = 2 (2-3) | N/A | S = 23048708  p-value = <0.01**  rho = 0.475 | NA |  |
| **5.2.6 Anticipated effect on the correctness of data collected in CT (Mean (SD); Med (IQR))**   - Much less correct [0] – much more correct [4] (5-point Likert scale) | Mean (SD) = 1.86 (0.89)  Med (IQR) = 2 (1-2) | N/A | S = 24708052  p-value = <0.01**  rho = 0.437 | N/A |  |
| **5.2.7. Anticipated effect on the completeness of data collected in CT (Mean (SD); Med (IQR))**   - Much less complete [0] – much more complete [4] (5-point Likert scale) | Mean (SD) = 1.82 (0.92)  Med (IQR) = 2 (1-2) | N/A | S = 25060878  p-value = <0.01**  rho = 0.429 | N/A |  |
| **5.2.8. Anticipated effect on the control of PHPs over the CT-process (Mean (SD); Med (IQR))**   - Much less control [0] – much more control [4] (5-point Likert scale) | Mean (SD) = 1.6 (0.94)  Med (IQR) = 2 (1-2) | N/A | S = 25429958  p-value = <0.01**  rho = 0.421 | N/A |  |
| **5.2.9. Anticipated willingness of cases to participate in CT with DCTS-tool (Mean (SD); Med (IQR))**   - Insufficiently willing [0] – sufficiently willing [4] (5-point Likert scale) | Mean (SD) = 2.17 (0.9)  Med (IQR) = 2 (2-3) | N/A | S = 22356885  p-value = <0.01**  rho = 0.491 | N/A |  |
| **Anticipated willingness of contacts to participate in CT with DCTS-tool (Mean (SD); Med (IQR))**   - Insufficiently willing [0] – sufficiently willing [4] (5-point Likert scale) | N/A | N/A | N/A | N/A |  |
| **5.2.10. Anticipated skills of cases to participate in CT with DCTS-tool (Mean (SD); Med (IQR))**   - Insufficiently skilled [0] – sufficiently skilled [4] (5-point Likert scale) | Mean (SD) = 2.21 (0.82)  Med (IQR) = 2 (2-3) | N/A | S = 25474129  p-value = <0.01**  rho = 0.42 | N/A |  |
| **Anticipated skills of contacts to participate in CT with DCTS-tool (Mean (SD); Med (IQR))**   - Insufficiently skilled [0] – sufficiently skilled [4] (5-point Likert scale) | N/A | N/A | N/A | N/A |  |
| **Anticipated effect on the number of identified cases, settings, and clusters in CT (Mean (SD); Med (IQR))**   - Much less [0] – much more [4] (5-point Likert scale) | N/A | N/A | N/A | N/A |  |
| **5.2.11.** **Anticipated effect on the number of contacts reached in CT (Mean (SD); Med (IQR))**   - Much less [0] – much more [4] (5-point Likert scale) | Mean (SD) = 2.07 (1.04)  Med (IQR) = 2 (1-3) | N/A | S = 25189318  p-value = <0.01**  rho = 0.426 | N/A |  |
| **5.2.12.** **Anticipated effect on adequately informing contacts (Mean (SD); Med (IQR))**   - Much worse [0] – much better [4] (5-point Likert scale) | Mean (SD) = 2 (0.96)  Med (IQR) = 2 (1-3) | N/A | S = 22497357  p-value = <0.01**  rho = 0.487 | N/A |  |
| **5.2.13. Anticipated effect on the support that PHPs can offer to cases (Mean (SD); Med (IQR))**   - Much worse [0] – much better [4] (5-point Likert scale) | Mean (SD) = 2.25 (0.83)  Med (IQR) = 2 (2-3) | N/A | S = 20978649  p-value = <0.01**  rho = 0.522 | N/A |  |
| **5.2.14. Anticipated effect on the support that PHPs can offer to contacts (Mean (SD); Med (IQR))**   - Much worse [0] – much better [4] (5-point Likert scale) | Mean (SD) = 2.03 (0.97)  Med (IQR) = 2 (1-3) | N/A | S = 24024171  p-value = <0.01**  rho = 0.453 | N/A |  |
| **Anticipated effect on compliance of cases with CT-measures and guidelines (Mean (SD); Med (IQR))**   - Much worse [0] – much better [4] (5-point Likert scale) | N/A | N/A | N/A | N/A |  |
| **5.2.15. Anticipated effect on compliance of contacts with CT-measures and guidelines (Mean (SD); Med (IQR))**   - Much worse [0] – much better [4] (5-point Likert scale) | Mean (SD) = 1.82 (0.81)  Med (IQR) = 2 (1-2) | N/A | S = 23845160  p-value = <0.01**  rho = 0.457 | N/A |  |
| QUESTIONNAIRE SECTION 5: BELIEFS REGARDING BENEFITS AND CHALLENGES OF DCTS-TOOL 3 | | | | | |
| Questionnaire item | Individuals who completed the online questionnaire (N= 641) | Bivariate association with intention to use DCTS-tool 1 (N/A) | Bivariate association with intention to use DCTS-tool 2 (N/A) | Bivariate association with intention to use DCTS-tool 3 |  |
| **5.3.1. Anticipated effect on speed of CT** (Mean (SD); Med (IQR))   - Much slower [0] – much faster [4] (5-point Likert scale) | Mean (SD) = 2.73 (0.8)  Med (IQR) = 3 (2-3) | N/A | N/A | S = 27160557  p-value = <0.01**  rho = 0.381 |  |
| **5.3.2. Anticipated effect on the workload of PHPs in CT** (Mean (SD); Med (IQR))   - Much more work [0] – much less work [4] (5-point Likert scale) | Mean (SD) = 2.83 (0.88)  Med (IQR) = 3 (2-3) | N/A | N/A | S = 31847757  p-value = <0.01**  rho = 0.274 |  |
| **5.3.3. Anticipated effect on the difficulty of CT** (Mean (SD); Med (IQR))   - Much more difficult [0] – much easier [4] (5-point Likert scale) | Mean (SD) = 2.67 (0.81)  Med (IQR) = 3 (2-3) | N/A | N/A | S = 25715173  p-value = <0.01**  rho = 0.414 |  |
| **Anticipated effect on the pleasantness to participate in CT for cases** (Mean (SD); Med (IQR))   - Much less pleasant [0] – much more pleasant [4] (5-point Likert scale) | N/A | N/A | N/A | N/A |  |
| **5.3.4. Anticipated effect on the pleasantness to participate in CT for contacts** (Mean (SD); Med (IQR))   - Much less pleasant [0] – much more pleasant [4] (5-point Likert scale) | Mean (SD) = 1.95 (0.93)  Med (IQR) = 2 (1-3) | N/A | N/A | S = 18560386  p-value = <0.01**  rho = 0.577 |  |
| **5.3.5. Anticipated effect on the correctness of data collected in CT (Mean (SD); Med (IQR))**   - Much less correct [0] – much more correct [4] (5-point Likert scale) | Mean (SD) = 1.94 (0.99)  Med (IQR) = 2 (1-3) | N/A | N/A | S = 22766116  p-value = <0.01**  rho = 0.481 |  |
| **5.3.6. Anticipated effect on the completeness of data collected in CT (Mean (SD); Med (IQR))**   - Much less complete [0] – much more complete [4] (5-point Likert scale) | Mean (SD) = 1.99 (1.05)  Med (IQR) = 2 (1-3) | N/A | N/A | S = 21276674  p-value = <0.01**  rho = 0.515 |  |
| **5.3.7. Anticipated effect on the control of PHPs over the CT-process (Mean (SD); Med (IQR))**   - Much less control [0] – much more control [4] (5-point Likert scale) | Mean (SD) = 1.65 (0.96)  Med (IQR) = 2 (1-2) | N/A | N/A | S = 26165540  p-value = <0.01**  rho = 0.404 |  |
| **Anticipated willingness of cases to participate in CT with DCTS-tool (Mean (SD); Med (IQR))**   - Insufficiently willing [0] – sufficiently willing [4] (5-point Likert scale) | N/A | N/A | N/A | N/A |  |
| **5.3.8. Anticipated willingness of contacts to participate in CT with DCTS-tool (Mean (SD); Med (IQR))**   - Insufficiently willing [0] – sufficiently willing [4] (5-point Likert scale) | Mean (SD) = 1.88 (0.94)  Med (IQR) = 2 (1-3) | N/A | N/A | S = 17001676  p-value = <0.01**  rho = 0.613 |  |
| **Anticipated skills of cases to participate in CT with DCTS-tool (Mean (SD); Med (IQR))**   - Insufficiently skilled [0] – sufficiently skilled [4] (5-point Likert scale) | N/A | N/A | N/A | N/A |  |
| **5.3.9. Anticipated skills of contacts to participate in CT with DCTS-tool (Mean (SD); Med (IQR))**   - Insufficiently skilled [0] – sufficiently skilled [4] (5-point Likert scale) | Mean (SD) = 2.18 (0.83)  Med (IQR) = 2 (2-3) | N/A | N/A | S = 24021933  p-value = <0.01**  rho = 0.453 |  |
| **Anticipated effect on the number of identified cases, settings, and clusters in CT (Mean (SD); Med (IQR))**   - Much less [0] – much more [4] (5-point Likert scale) | N/A | N/A | N/A | N/A |  |
| **Anticipated effect on the number of contacts reached in CT (Mean (SD); Med (IQR))**   - Much less [0] – much more [4] (5-point Likert scale) | N/A | N/A | N/A | N/A |  |
| **Anticipated effect on**  **te notification/informing of contacts (Mean (SD); Med (IQR))**   - Much worse [0] – much better [4] (5-point Likert scale) | N/A | N/A | N/A | N/A |  |
| **Anticipated effect on the support that PHPs can offer to cases (Mean (SD); Med (IQR))**   - Much worse [0] – much better [4] (5-point Likert scale) | N/A | N/A | N/A | N/A |  |
| **5.3.10. Anticipated effect on the support that PHPs can offer to contacts (Mean (SD); Med (IQR))**   - Much worse [0] – much better [4] (5-point Likert scale) | Mean (SD) = 2.15 (0.99)  Med (IQR) = 2 (1-3) | N/A | N/A | S = 22035770  p-value = <0.01**  rho = 0.498 |  |
| **Anticipated effect on compliance of cases with CT-measures and guidelines (Mean (SD); Med (IQR))**   - Much worse [0] – much better [4] (5-point Likert scale) | N/A | N/A | N/A | N/A |  |
| **5.3.11. Anticipated effect on compliance of contacts with CT-measures and guidelines (Mean (SD); Med (IQR))**   - Much worse [0] – much better [4] (5-point Likert scale) | Mean (SD) = 2.02 (0.89)  Med (IQR) = 2 (1-3) | N/A | N/A | S = 20830755  p-value = <0.01**  rho = 0.525 |  |

* Statistically significant at alpha = 0.05

** Statistically significant at alpha = 0.01

***Category not included in bivariate analysis due to few observations

**Table C.** **Bivariate associations between PHPs’ demographic characteristics and questionnaire completion (yes/no) to assess if respondent dropout biased our study.** Several characteristics are statistically significantly associated with questionnaire completion. Effect sizes can typically be categorized as small or medium.

| Characteristic | Questionnaire completion | | Test, test statistic,  degrees of freedom | P-value of association | Effect size |
| --- | --- | --- | --- | --- | --- |
|  | Yes (N=641) | No (N=221) |  |  |  |
| Age (Mean (SD)) | 40.7 (15.3) | 36.5 (14.3) | T-test, t=-3.67, df=408.28 | <0.01* | cohen’s d = -0.28 (small) |
| Gender (%)   - Female - Male - Non-binary** | 462 (72.1)  177 (27.6)  2 (0.3) | 162 (73.3)  57 (25.8)  2 (0.9) | Chi^2^, X^2^=0.15, df=1, | 0.70 | Phi = 0.02 (small) |
| Province of employment (%)   - Drenthe** - Flevoland** - Friesland - Gelderland - Groningen - Limburg - Noord-Brabant - Noord-Holland - Overijssel - Utrecht - Zeeland - Zuid-Holland - Multiple provinces** | 4 (0.6)  15 (2.3)  71 (11.1)  143 (22.3)  27 (4.2)  58 (9.0)  37 (5.8)  71 (11.1)  70 (10.9)  57 (8.9)  34 (5.3)  50 (7.8)  4 (0.6) | 1 (0.5)  4 (1.8)  31 (14.0)  42 (19.0)  7 (3.2)  25 (11.3)  20 (9.0)  23 (10.4)  16 (7.2)  24 (10.9)  6 (2.7)  21 (9.5)  1 (0.5) | Chi^2^, X^2^=11.90, df=9 | 0.22 | Cramer’s V = 0.12 (medium) |
| Primary occupation at PHS (%)   - CT-manager/coordinator - Temporary contact tracer - PHS-physician - PHS-nurse - Other** | 25 (3.9)  538 (83.9)  39 (6.1)  32 (5.0)  7 (1.1) | 18 (8.1)  154 (69.7)  20 (9.0)  27 (12.2)  2 (0.9) | Chi^2^, X^2^=24.71, df=3 | <0.01* | Cramer’s V = 0.17 (medium) |
| Experience with CT for COVID-19 (%)   - Other CT experience than COVID-19** - <1 month** - 1-6 months - 7-12 months - 1-2 years | 15 (2.3)  13 (2.0)  383 (59.8)  183 (28.5)  92 (14.4) | 6 (2.7)  4 (1.8)  127 (57.5)  41 (18.6)  43 (19.5) | Chi^2^, X^2^=3.58, df=2 | 0.17 | Cramer’s V = 0.06 (small) |
| Experience with CT for communicable diseases (other than COVID-19) (%)   - Yes - No | 53 (8.3)  588 (91.7) | 31 (14.0)  190 (86.0) | Chi^2^, X^2^=5.56, df=1 | 0.02* | Phi = 0.08 (small) |

* Statistically significant at alpha = 0.05; ** Category not included in bivariate analysis because at least one expected bin size was below 5

**Figure A. Correlation matrix for intention to use DCTS-tool 1 in CT for COVID-19. Includes all questionnaire items, except socio demographic characteristics (i.e., sections 2-5 are included)**

**
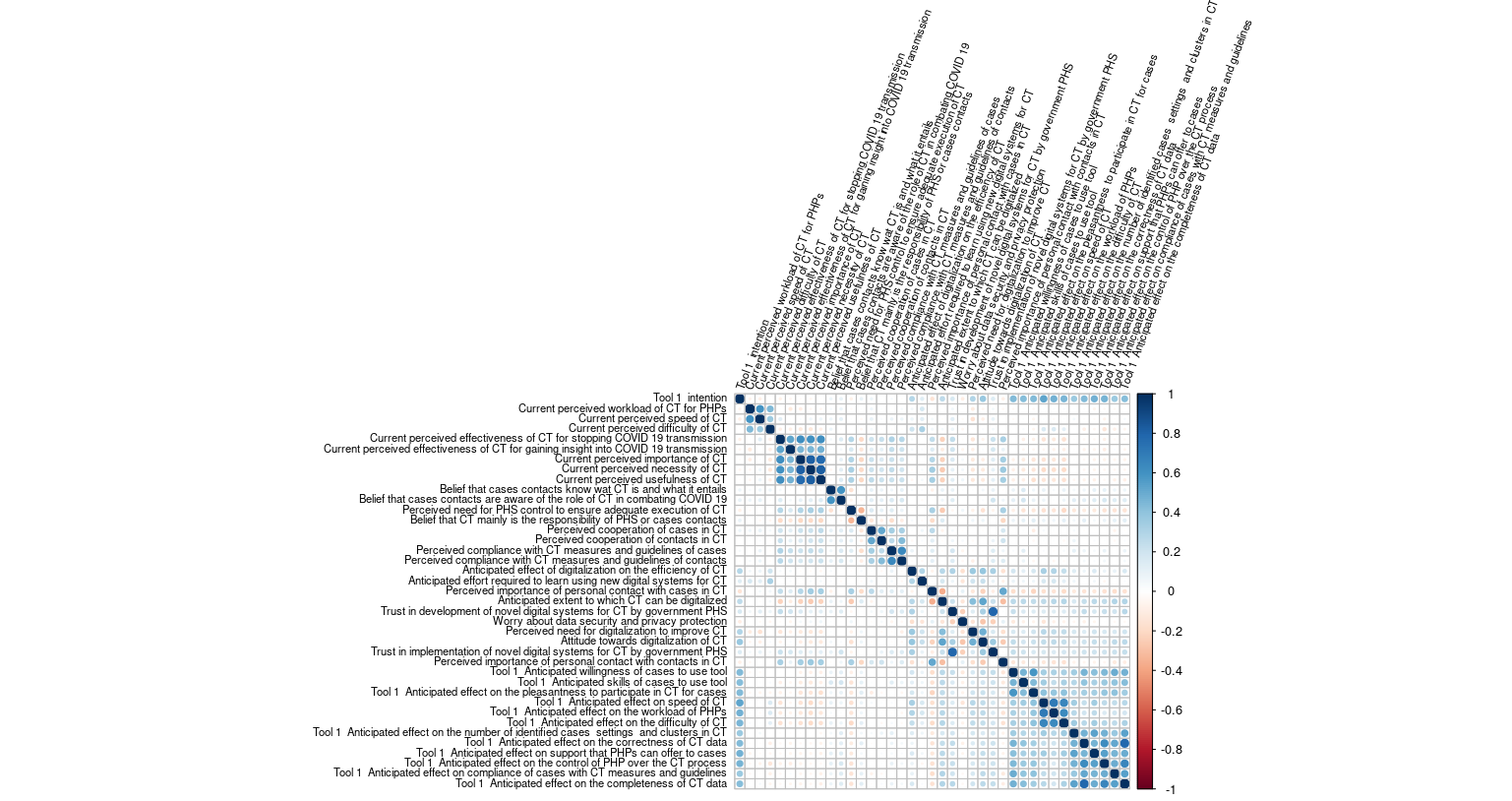
**

**Figure B. Correlation matrix for intention to use DCTS-tool 2 in CT for COVID-19.** Includes all questionnaire items, except socio demographic characteristics (i.e., sections 2-5 are included)


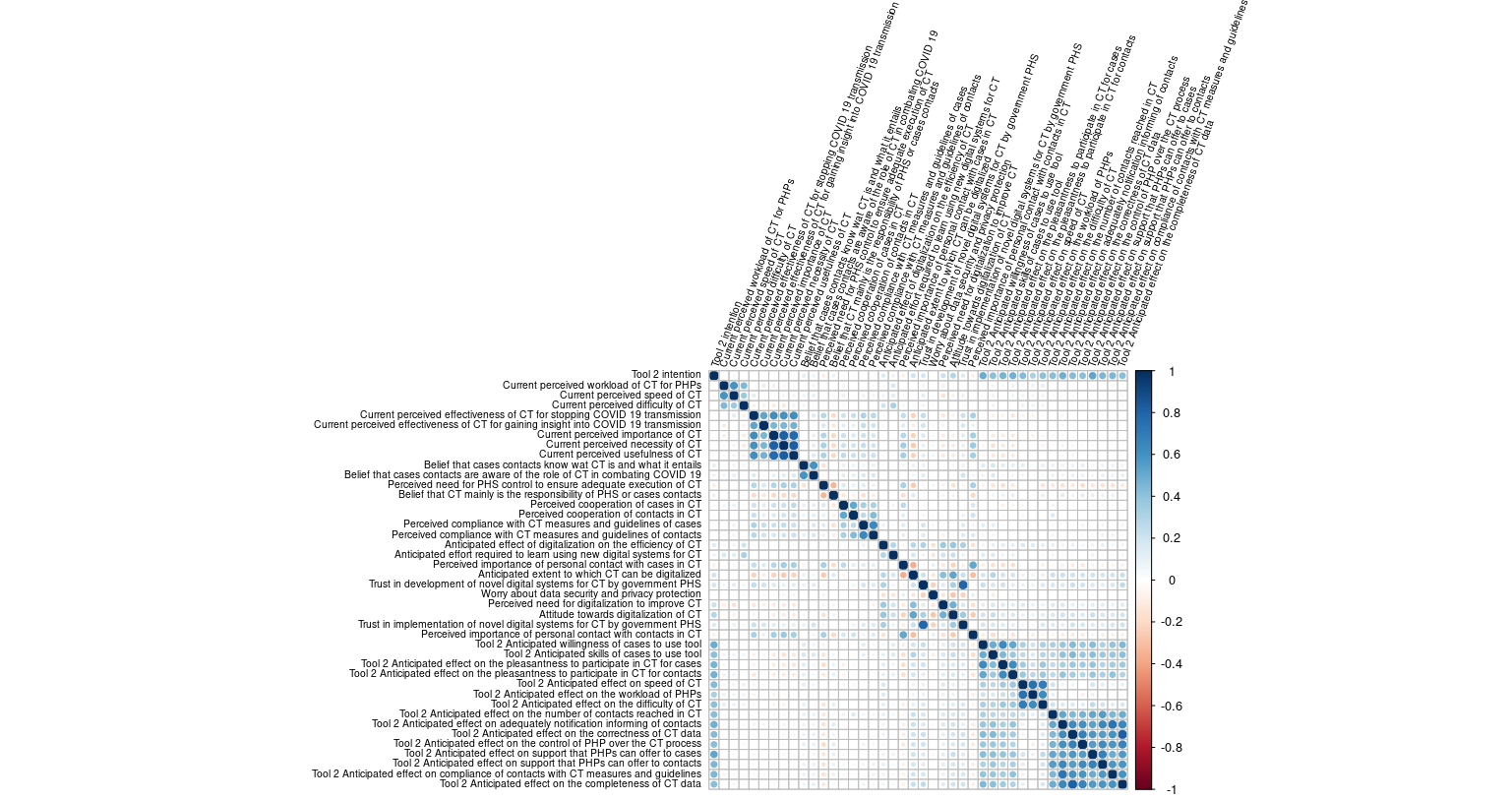


**Figure C. Correlation matrix for intention to use DCTS-tool 3 in CT for COVID-19.** Includes all questionnaire items, except socio demographic characteristics (i.e., sections 2-5 are included)


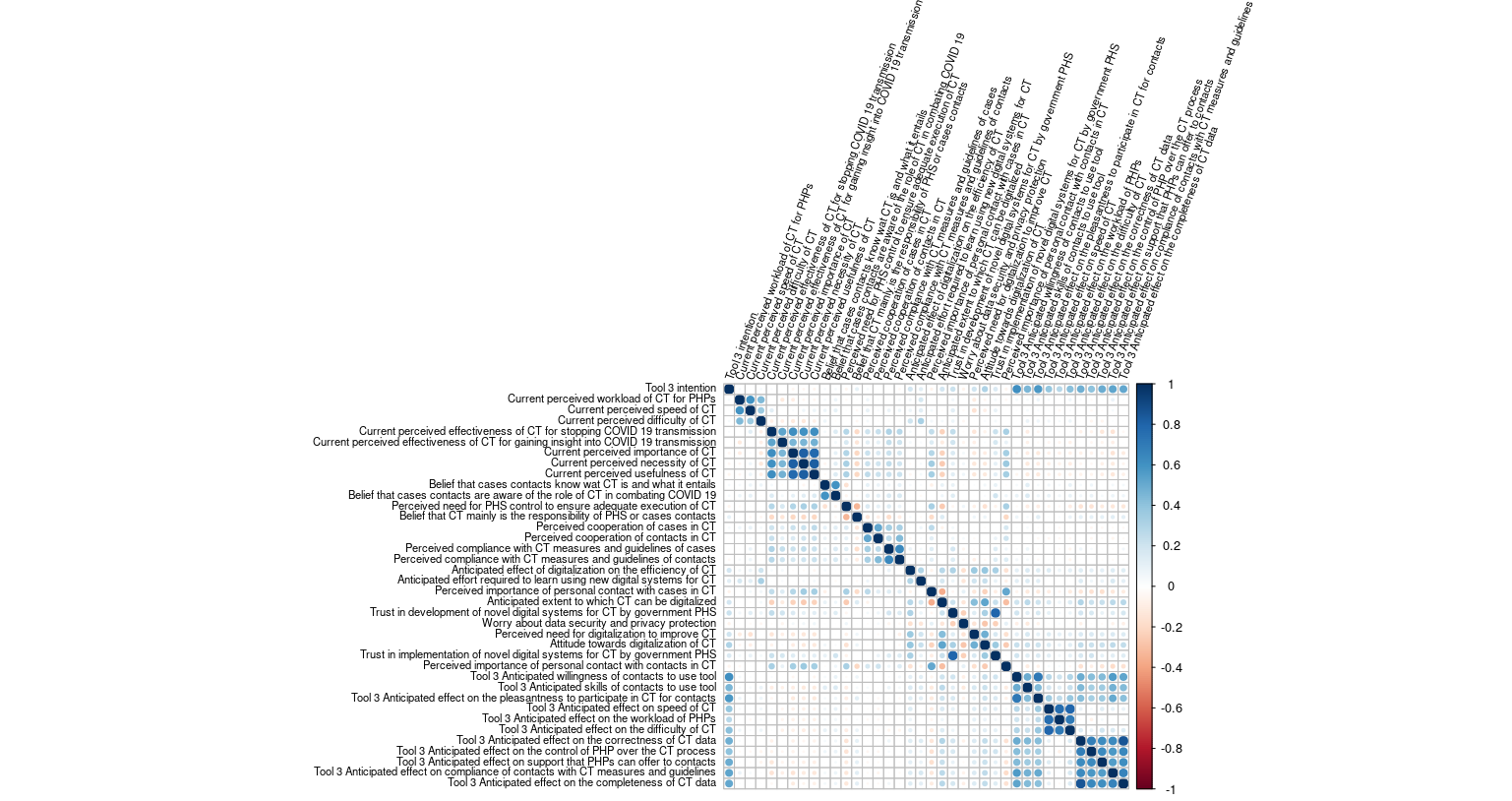


**Figure D. Cluster dendrogram of determinants, as identified in the VIR of DCTS-tool 1.** The y-axis shows the ‘distance’ (i.e., 1 - the average absolute correlation) between individual and/or clusters of variables. The red line references a correlation of 0.5. Variables that are more closely linked together lower in the dendrogram are more closely/strongly correlated and vice versa. We distinguished and named 4 clusters: Cluster 1. ‘Feasibility and efficiency of CT’; Cluster 2. ‘Support for cases and contacts to adequately perform CT’; Cluster 3. ‘Willingness, preferences and skills of cases and contacts’; and Cluster 4. ‘Digitalization of CT’. Cluster analyses were performed using the ‘klaR’ package in R.


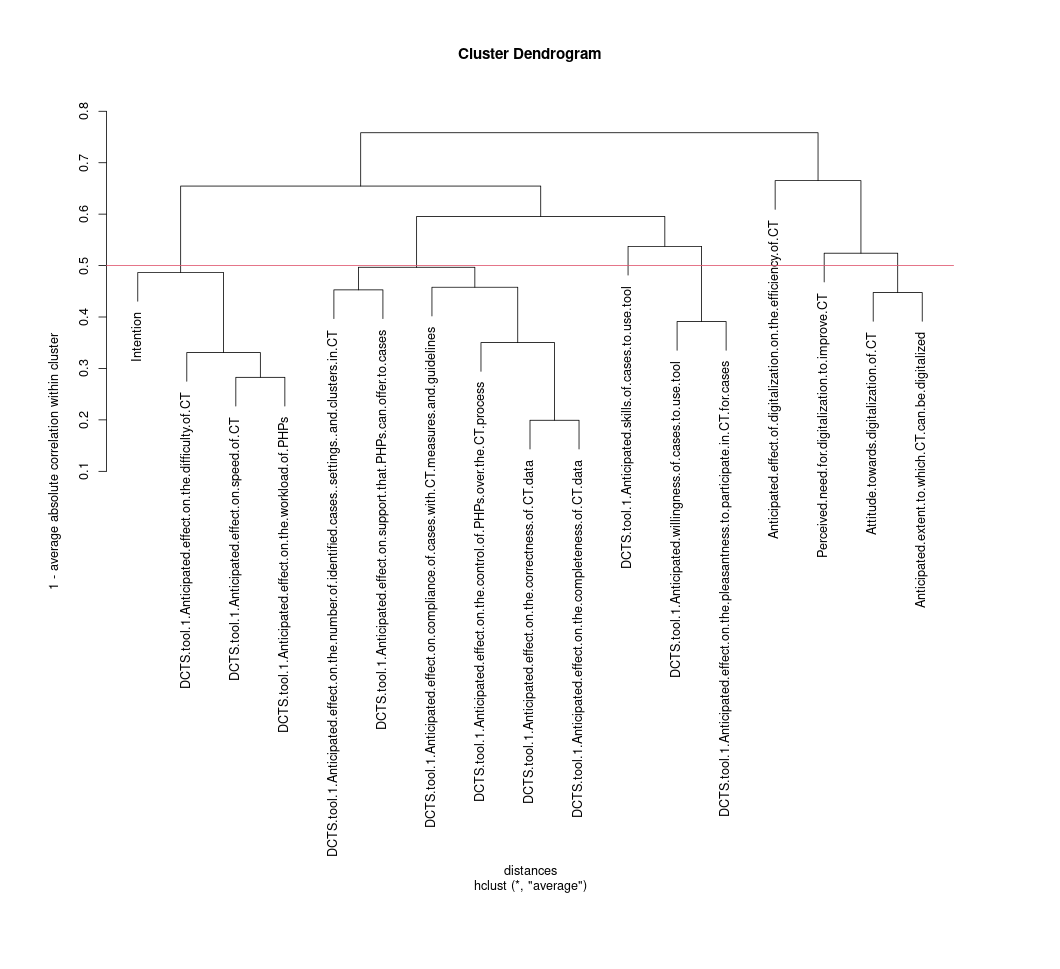


**Cluster 1.**

**Cluster 2.**

**Cluster 3.**

**Cluster 4.**

**
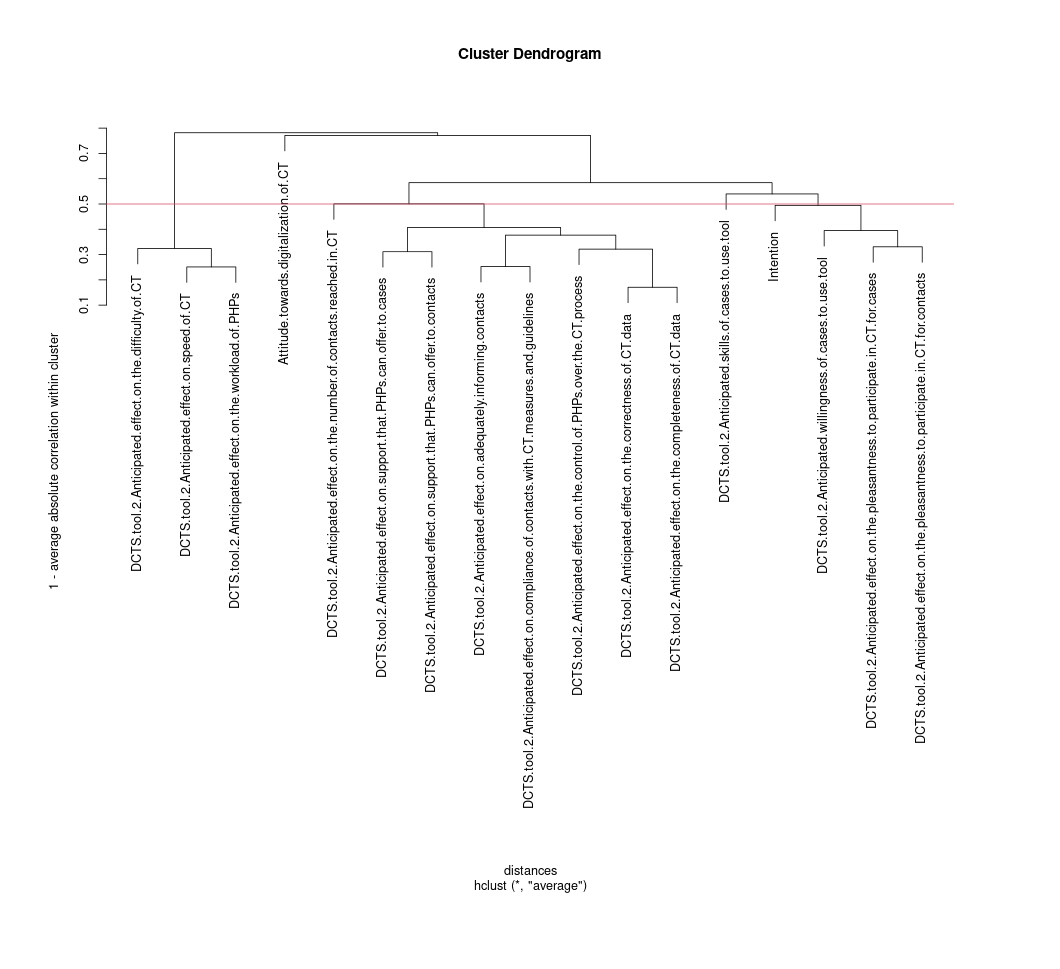
Figure E. Cluster dendrogram of determinants, as identified in the VIR of DCTS-tool 2.** The y-axis shows the ‘distance’ (i.e., 1 - the average absolute correlation) between individual and/or clusters of variables. The red line references a correlation of 0.5. Variables that are more closely linked together lower in the dendrogram are more closely/strongly correlated and vice versa. We distinguished and named 4 clusters: Cluster 1. ‘Feasibility and efficiency of CT’; Cluster 2. ‘Support for cases and contacts to adequately perform CT’; Cluster 3. ‘Willingness, preferences and skills of cases and contacts’; and Cluster 4. ‘Digitalization of CT’. Cluster analyses were performed using the ‘klaR’ package in R.

**Cluster 4.**

**Cluster 3.**

**Cluster 2.**

**Cluster 1.**

**
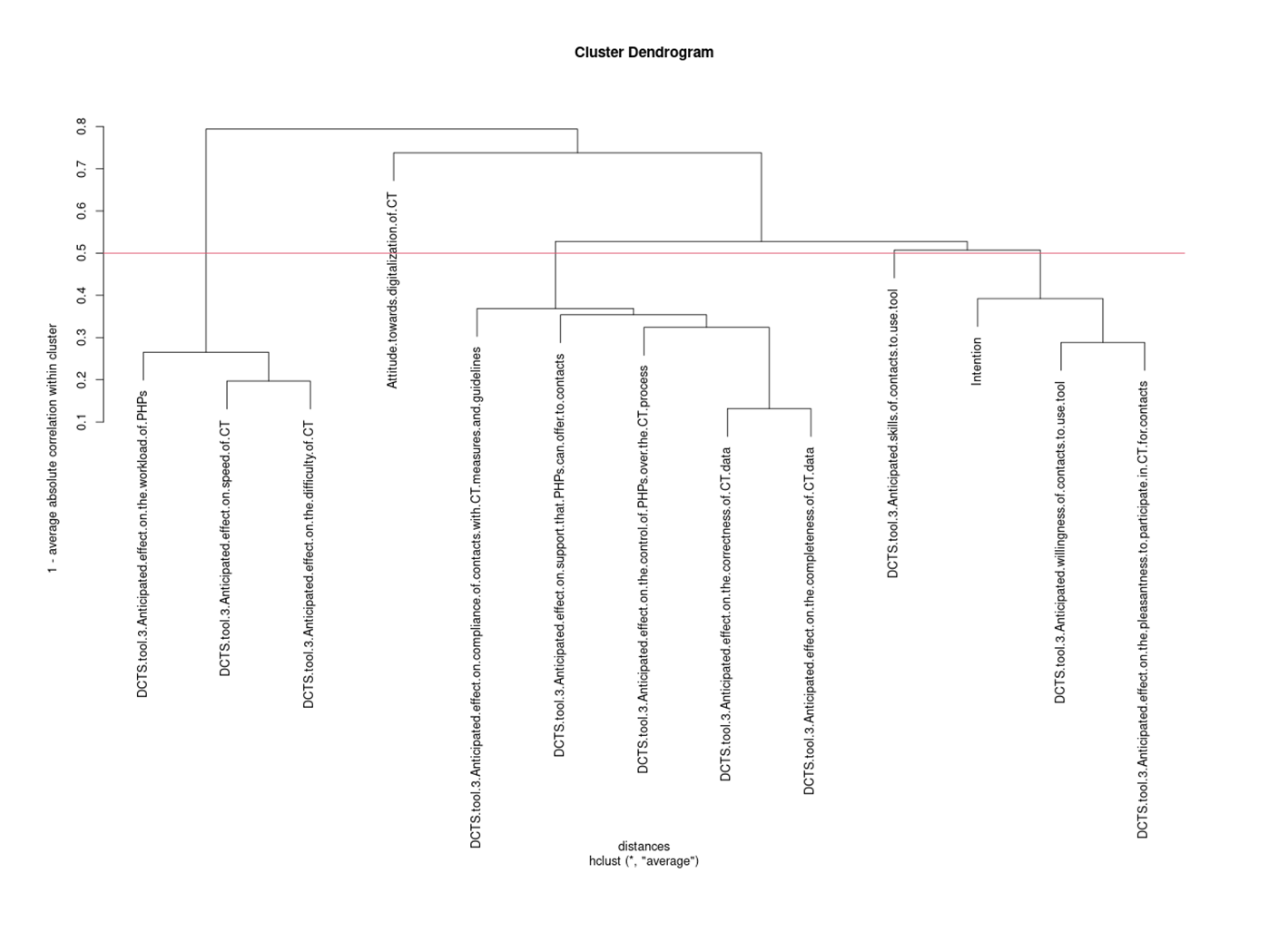
Figure F. Cluster dendrogram of determinants, as identified in the VIR of DCTS-tool 3.** The y-axis shows the ‘distance’ (i.e., 1 - the average absolute correlation) between individual and/or clusters of variables. The red line references a correlation of 0.5. Variables that are more closely linked together lower in the dendrogram are more closely/strongly correlated and vice versa. We distinguished and named 4 clusters: Cluster 1. ‘Feasibility and efficiency of CT’; Cluster 2. ‘Support for cases and contacts to adequately perform CT’; Cluster 3. ‘Willingness, preferences and skills of cases and contacts’; and Cluster 4. ‘Digitalization of CT’. Cluster analyses were performed using the ‘klaR’ package in R.

**Cluster 4.**

**Cluster 3.**

**Cluster 2.**

**Cluster 1.**

**Fig G. Variable importance ranking in relation to PHPs’ intention to use DCTS-tool 1, including questionnaire completion as a predictor to assess if respondent dropout biased our results.** This plot was produced through a random forest analysis that we performed on a dataset in which we included all respondents who at had at least answered all questions pertaining to DCTS-tool 1 (N=702). In the analysis, we included a variable measuring whether these respondents thereafter proceeded to complete the full questionnaire (yes/no) as a predictor of PHPs’ intention, to assess if respondent dropout may have biased our analyses. The variable ‘Questionnaire.completion’ is located to the left of the graph, indicating that respondent dropout had a negligible effect on our results.

**
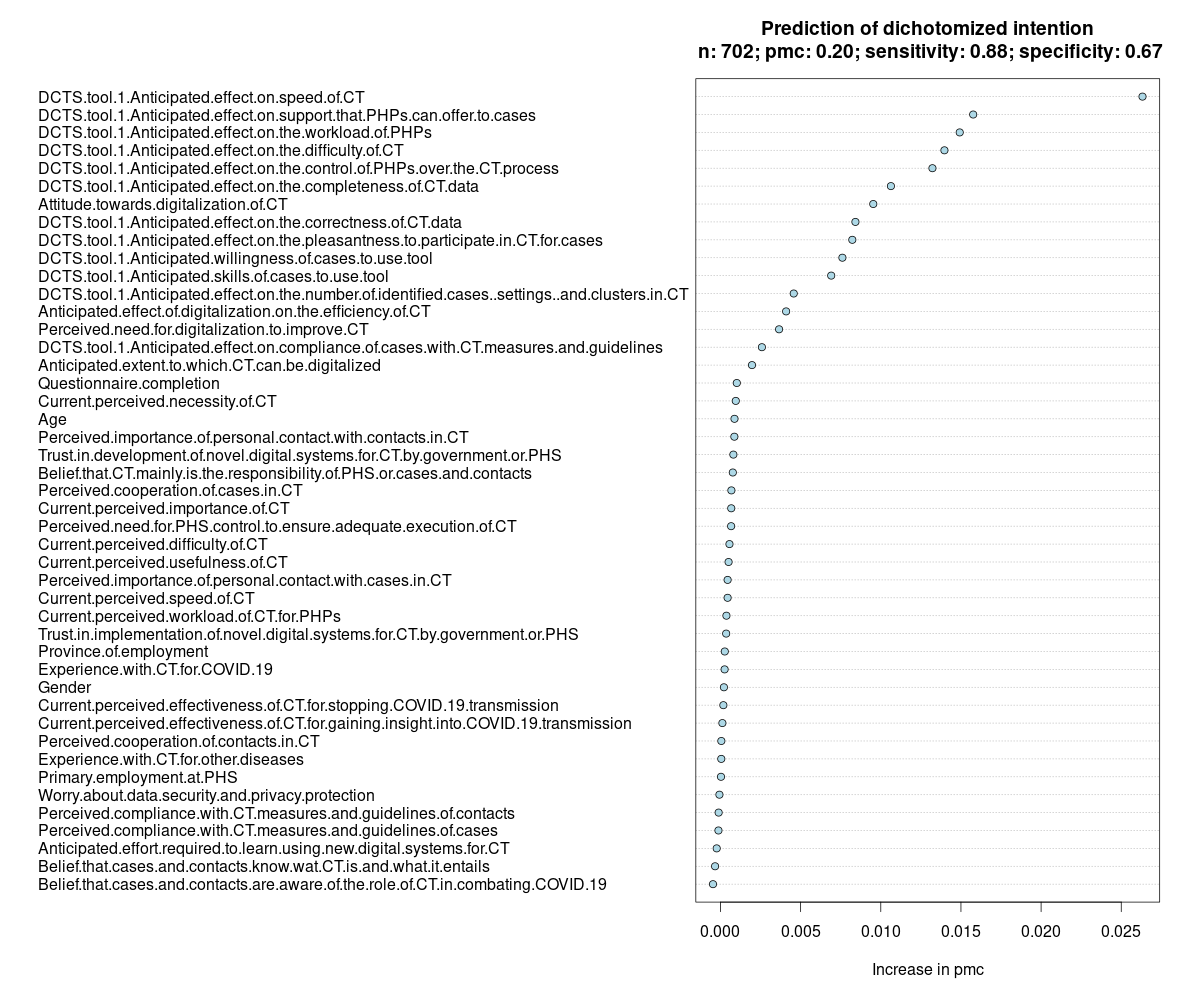
**

**Figure H. ROC analysis for RF-model DCTS-tool 1**


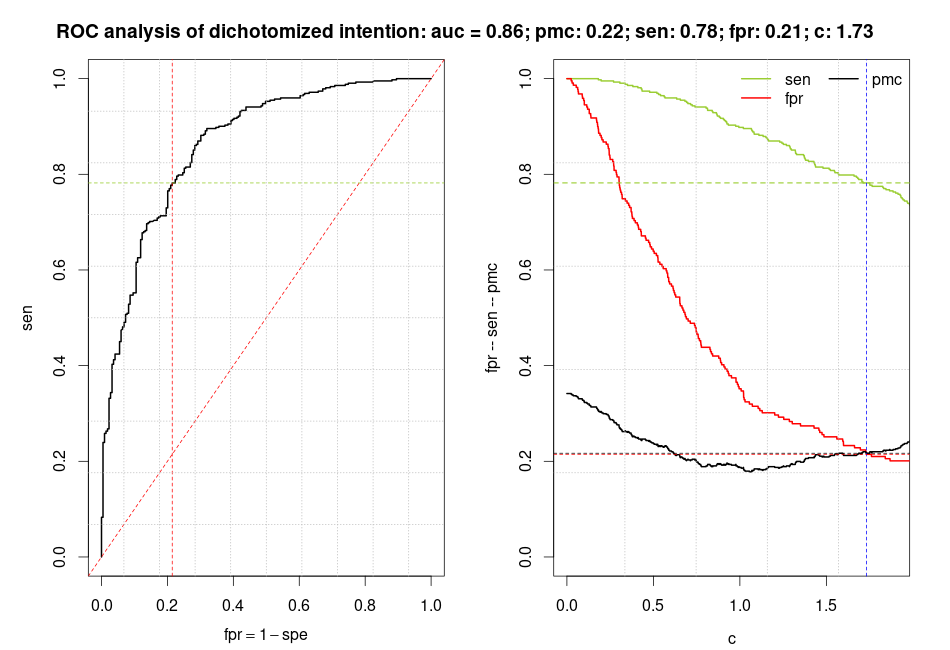


**Figure I. ROC analysis for RF-model DCTS-tool 2**


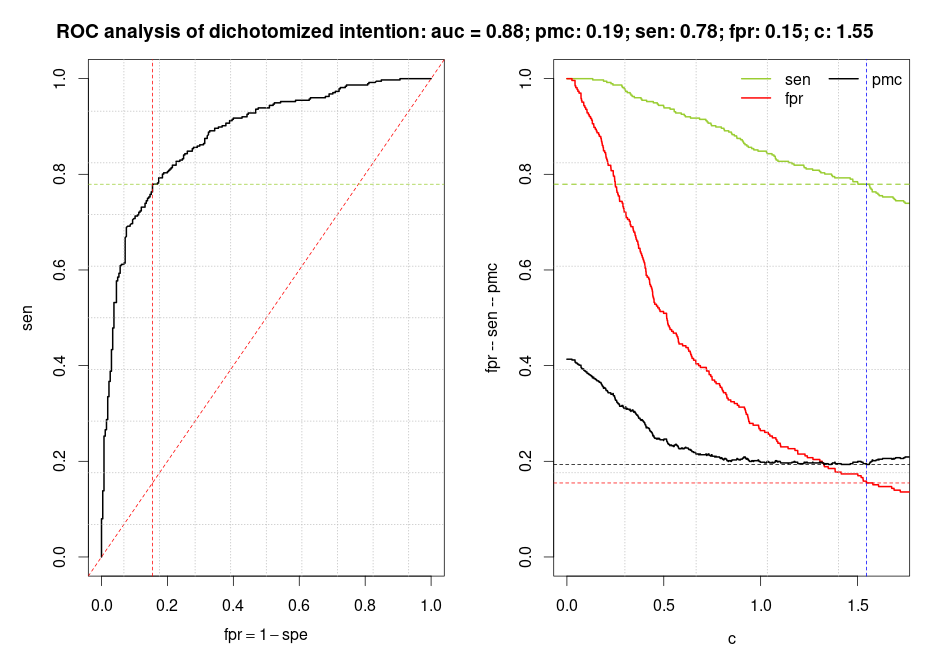


**Figure J. ROC analysis for RF-model DCTS-tool 3**
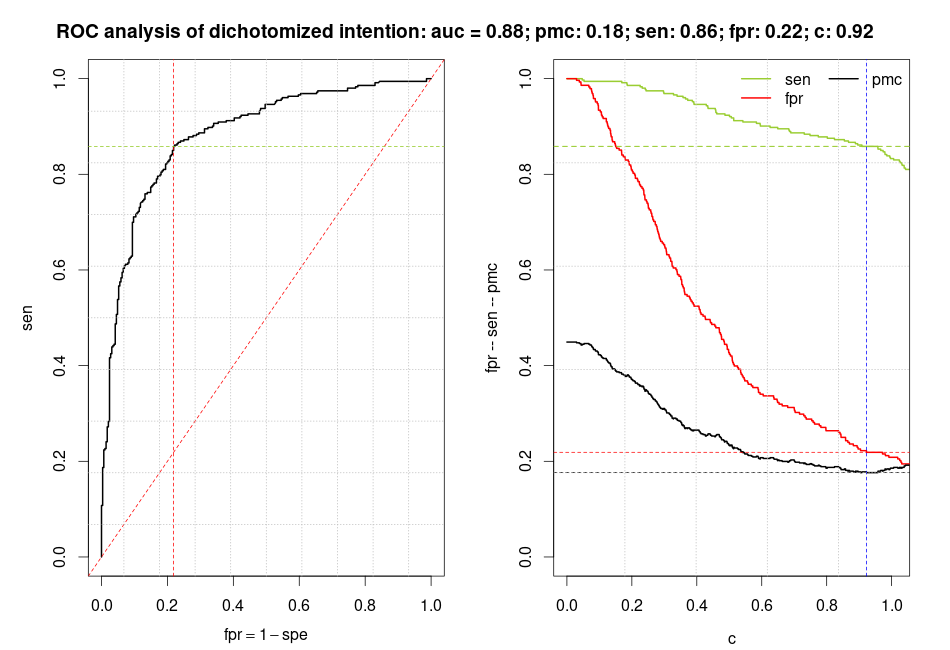

Supplement: S3 Appendix — (DOCX) [file pdig.0000425.s003.docx]
