# Supplementary material for: Determinants of Dutch public health professionals’ intention to use digital contact tracing support tools: A cross-sectional online questionnaire study
Source: PLOS Digit Health. 2024 Feb 14;3(2):e0000425. doi: 10.1371/journal.pdig.0000425 (PMC10866487; doi:10.1371/journal.pdig.0000425)
Supplement: Attachment — Submitted filename: Response to Reviewers.docx [file pdig.0000425.s005.docx]

National Institute for Public Health and the Environment

Centre for Infectious Disease Control

National Coordination Centre for Communicable Disease Control

Antonie van Leeuwenhoeklaan 9

3720 BA Bilthoven

The Netherlands

[yannick.helms@rivm.nl](mailto:yannick.helms@rivm.nl)

19^th^ of October 2023

Dear Haleh Ayatollahi,

We are very happy to hear that you are interested in publishing our manuscript in PLOS Digital Health. Many thanks to you and the three reviewers for your valuable suggestions to improve our manuscript titled ‘Determinants of Dutch public health professionals’ intention to use digital contact tracing support tools: a cross-sectional online questionnaire’.

Based on the suggestions we received, we revised and improved the manuscript. Attached you can find our point-by-point response (in blue) to the comments of the three reviewers and the corresponding changes made in the manuscript.

We sincerely hope that our revised manuscript can be accepted for publication in PLOS Digital Health. All authors have seen and approved the revised manuscript.

Yours faithfully,

Yannick Helms, M.Sc.

(Corresponding author)

**REVIEWER 1:**

We would like to sincerely thank Shoshana Bloom for the elaborate and valuable suggestions to improve our manuscript. Our responses can be found below.

**COMMENT 1: Regarding your previous research, can you add the sample size of your previous research to support your justification for this further research with additional survey size.**

Amended. We included the sample size of our previous research in the manuscript (L109 - 115):

*‘We, therefore, previously conducted two studies among Dutch PHPs (17, 18). In the first study, we used interviews (N=12) and online questionnaires (N=70) to explore the opportunities and challenges of digitally involving cases and their contacts in CT under different circumstances (i.e., in the context of various close-contact pathogens and outbreak scenarios). In the second study, we performed interviews (N=17) focused specifically on exploring PHPs’ perspectives and needs regarding the application of three types of DCTS-tools in the context of the SARS-CoV-2 (COVID-19) pandemic, one for each stage of the CT-process (i.e., contact identification, notification, and monitoring).’*

**COMMENT2: I suggest the authors identify and present their research objectives, hypothesis and the question / questions that your survey was designed to answer. The research questions should then flow through the results and into the discussion sections.**

Amended. We now present the research question that guided our study explicitly at the end of the introduction - and answer it in the discussion section:

Introduction (L137 - 141): *‘We, therefore, performed an online questionnaire study among PHPs involved in the execution of CT for COVID-19 in the Netherlands, to improve our understanding of the determinants of PHPs’ intention to use DCTS-tools. The research question that guided this study was: ‘What are the determinants of Dutch PHPs’ intention to involve cases and their contacts in the identification, notification, and monitoring stages of the COVID-19 CT process, using DCTS-tools?’*

Discussion (L465 - 473): *‘**Most PHPs have a positive intention towards using DCTS-tools to involve cases and their contacts in all stages of the CT-process (i.e., contact identification, notification, and monitoring) for COVID-19. Although we observed some differences in the prioritization of individual determinants between the DCTS-tools, the overall groups of variables that determines PHPs’ intention to use them appears very similar. In general, important determinants of a positive intention among PHPs are the anticipated effect of DCTS-tools on the feasibility and efficiency of CT (speed, workload, difficulty), the degree to which PHPs anticipated that cases and contacts may find it pleasant and may be willing to participate in CT using DCTS-tools, and the degree to which PHPs anticipated that they could sufficiently support cases and contacts when using DCTS-tools.’*

In addition, we changed some terminology throughout the manuscript to align with our research question more accurately (e.g., we changed ‘predictor’ to ‘determinant’ where possible). We also changed several headings in the methods and results section to align with the main research question more clearly. For example, to make explicit that our multivariable analyses were aimed at identifying determinants of PHPs’ intention to use DCTS-tools, we changed the header *‘Multivariable analyses’* (in the methods section) to *‘Multivariable analyses: identifying determinants of PHPs’ intention to use DCTS-tools’* (L390 - 391).

**COMMENT 3: I suggest the authors expand the introduction to include more information about the study setting(s) - to more effectively set the scene for the methods section.**

Amended. We elaborate more on the study setting by providing more information on the COVID-19 situation and the way contact tracing was performed in the Netherlands at the time that this study was conducted. We included this information at the beginning of the methods section, under the header ‘Setting and study population’ (L148 - 159):

*‘CT in the Netherlands is usually performed at PHS, by specialized nurses under supervision of physicians. There are 25 PHS in the Netherlands, each representing a different geographical region.*

*This study was performed between February and April 2022. At that time, the number of newly identified COVID-19 cases per day was particularly high in the Netherlands, but regulations concerning contact restrictions to reduce transmission of COVID-19 (e.g., social distancing, maximum group sizes) were gradually being lifted due to increasing vaccination rates, diminishing hospitalizations rates of COVID-19 patients and the emergence of less severe variants of the virus. CT was still implemented on a large scale, but mostly in a (very) scaled-down manner. Therefore, PHS had temporarily hired many new contact tracers (e.g., medical students) and focused the responsibilities of nurses and physicians towards relatively complex clusters and the overall coordination of CT. Although national guidelines for CT were in place, the precise execution of CT (e.g., the degree to which CT was scaled down) often differed between PHS, depending on regional caseload and available personnel for CT.’*

**COMMENT 4: The authors might want to consider describing any efforts to address potential sources of bias and whether any sources of bias should be mentioned as a limitation.**

Amended. We now more elaborately describe how we aimed to address potential bias due to differences in CT-practices between PHS-regions and professionals backgrounds of respondents (in the section ‘Setting and study population’) by including respondents from all professional backgrounds and PHS-regions (L160 - 165):

*‘To minimize bias related to different CT practices between PHS and/or PHPs with different professional backgrounds and experience with CT, we included public health nurses and physicians, and temporary contact tracers (all of whom we refer to as PHPs) from all PHS in the Netherlands in our study. Eligibility criteria were: (1) being 18 years or older, (2) being or having been involved in the execution, coordination, and/or organization of CT for COVID-19 and/or other communicable diseases, and (3) currently working for a PHS in the Netherlands.’*

In addition, we included a paragraph in the methods section where we describe how we assessed potential bias in our study related to respondent dropout (i.e., respondents not finishing the questionnaire). We also included this analysis in S3 Appendix Table C and Figure G. This is described in the section ‘Assessment of bias due to respondent dropout’ (L311 - 325):

*‘**Since we only included individuals who completed the online questionnaire in our analyses, the results of this study may be biased if, for example, questionnaire completion was associated with certain demographic characteristics and/or with having a positive or negative intention towards the use of DCTS-tools. Therefore, to assess if respondent dropout may have biased our study results, we included a variable measuring questionnaire completion (yes/no) in the full dataset (also including individuals who did not complete the questionnaire) and computed bivariate associations between questionnaire completion and respondents’ demographics using chi-square and t-tests. In addition, we rebuilt the first RF-model (for DCTS-tool 1) with the full dataset, including the questionnaire completion variable as a predictor.*

*Although some demographic characteristics were statistically significantly associated with questionnaire completion, we did not find that the differences between the groups of individuals who completed the questionnaire and those who did not were meaningful/large. The additional RF-analysis indicated that questionnaire completion had a negligible effect on PHPs’ intention. This suggests that respondent dropout may have only limitedly influenced our results and findings. See also S3 Appendix Table C and Fig G’*

In the discussion section we also expanded on our previous assessment of potential bias (L549 - 565):

*‘Since we mainly relied on referrals and snowball sampling to distribute the online questionnaire, we were unable to calculate a response rate. In addition, we have no precise insights into characteristics of the overall population of PHPs at the time this study was conducted. This means that we cannot accurately estimate to what degree our study may have suffered from selection bias. Furthermore, our sample consisted mainly of temporary contact tracers, which may not represent the overall population of PHPs under ‘normal’ (i.e., non-pandemic) circumstances. This should be kept in mind when interpreting this study’s results and findings.*

*Nevertheless, we would like to emphasize that we managed to collect data from a large number of PHPs (N=641), representing all relevant socio-demographic and professional backgrounds as described in Table 1. Even though our sample consisted largely of temporary contact tracers, our RF-models show that PHPs’ socio-demographic and professional characteristics had a negligible effect on their intention to use DCTS-tools. This indicates that potential bias in our study related to over- or underrepresentation of professional backgrounds may be limited. As previously described, we also established that the potential bias in our study related to respondent dropout is limited. Therefore, we argue that we have obtained a good overall picture of PHPs’ attitudes and the factors influencing their intention to use DCTS-tools in the Netherlands, especially under pandemic circumstances and/or when large-scale CT is required.’*

**COMMENT 5: Were any survey responses incomplete and was any data cleansing performed?**

Amended: At the start of the results section, we already provided an overview of the number of individuals who started and finished the online questionnaire and were subsequently included in our analyses. To make it more explicit how we dealt with incomplete responses and to give more info on further preparation of the dataset, we included a new paragraph in the methods section named ‘Preparation of the dataset’ (L237 - 241):

*‘In our analyses, we only considered respondents who completed the online questionnaire, meaning they provided answers to all questions. Respondents who did not complete the questionnaire were discarded. Unnecessary columns, such as respondent ID’s and the dates and times of starting and finishing the questionnaire were also discarded. No further data cleaning was necessary/performed. ‘*

**COMMENT 6: As most of the respondents had only relatively little experience in CT - being temp workforce and no other experience with other diseases, do the authors feel this is a limitation that warrants including in the relevant section?**

Amended: We agree that this may have been a potential limitation of our study. However, our random forest analyses suggest that the influence of professional background (including also experience with other diseases) on the results and findings of our study was minimal/limited. In addition, our results are very much in line with previous research that we conducted among trained nurses and physicians only, which further supports this assessment. We now describe this more clearly in the discussion section, under the header ‘strengths and limitations’ (L558 - 561):

*‘Even though our sample consisted largely of temporary contact tracers, our RF-models show that PHPs’ socio-demographic and professional characteristics had a negligible effect on their intention to use DCTS-tools. This indicates that potential bias in our study related to over- or underrepresentation of professional backgrounds may be limited.’*

**COMMENT 7: The authors might want to consider whether reflecting on any differences between the results of the 3 tools would add value within the discussion**

Partially amended: Although we did observe differences in the prioritization of individual determinants of PHPs’ intention between the DCTS-tools, our results suggest that the same sets (or clusters) of variables can explain PHPs’ intention to use the different DCTS-tools to a similar degree. We now more explicitly state this in the beginning of the discussion section (L467 - 469):

*‘Although we observed some differences in the prioritization of individual determinants between the DCTS-tools, the overall groups of variables that determines PHPs’ intention to use them appears very similar.’*

In the ‘Recommendations’ section, we further argue that items should be considered as groups or ‘clusters’, due to their conceptual overlap, which is also reflected in the correlations between determinants. We, therefore, feel that it better fits our results and observations – and is more useful - to not elaborate further on the differences between DCTS-tools, but rather emphasize the similarities in terms of the described clusters. See also our response to comment 9, where we elaborate on this in more detail.

**COMMENT 8: The authors offer some suggestions for functionality based on the factors influencing intent to use DHTs of the respondents. Authors should make this clear how these recommendations were identified and whether this is the opinion of the authors or whether these recommendations have been tested or informed by other research.**

Amended: The recommendations are largely based on the involved researchers own expertise and observations and on previous qualitative research that we performed. We agree that this was not described clearly before and now more clearly describe that our recommendations are based on these sources (L591 - 594):

*‘In line with the identified clusters, we provide some elements to be considered for further implementation in practice. Note that these suggestions are based on our own personal insights and knowledge of behavior change methods (40), and insights from previous research that we conducted (17, 18).’*

**COMMENT 9: I suggest making the predictors of intent that were grouped together to be made clearer.**

Amended: We agree that the clustering of variables may have seemed somewhat arbitrary, or at least unclear. Therefore, to aid the interpretation of the clusters, make the clustering more explicit, and to ground the clustering more thoroughly in our data, we performed agglomerative hierarchical cluster analyses (HCA) on the determinants of each DCTS-tool (separately). HCA can be used as an exploratory method to compute correlations between (clusters) of variables, so to assess their ‘similarity’. The results of the HCA can be visualized in a ‘cluster dendrogram’, which allows for easy identification of groups, or clusters, of related variables. As such, HCA is also a useful tool to examine potential collinearity between variables. We now mention the HCA in the methods section, as part of our multicollinearity assessment under the header ‘Multivariable analyses: identifying determinants of PHPs’ intention to use DCTS-tools’ (L285 - 291):

*‘Instead of logistic or linear regression, we chose RF because of its flexibility in dealing with many variables (around 45 per model in our case) and its inherent greater ability to mimic the behavior of the data when based on a large sample (24). In addition, since we derived our questionnaire items from previously identified qualitative themes (meaning that many questionnaire items are conceptually related), we expected the predictors to be substantially correlated, which is problematic in regression analyses (25). See also Figs A, B, C, D, E and F in S3 Appendix, where we examined this in more detail using correlation matrices and agglomerative hierarchical cluster analyses (26).’*

As described in this section, we included the results of the HCA (the cluster dendrograms) that we used to identify/name the clusters in Figs D, E, and F in S3 Appendix. We now also state this in the discussion section ‘Recommendations’ to make it clear that the clustering is based on the HCA (L579 - 583):

*‘Based on our results we believe that there are three clusters of determinants that may be prioritized in the development and implementation of DCTS-tools for COVID-19. For more information on how we established/identified the clusters we refer to Figs D, E, and F in S3 Appendix, where we show the output of agglomerative hierarchical cluster analyses that we performed as part of our (multi)collinearity assessment.’*

**REVIEWER 2:**

Yes, this s manuscript meet PLOS Digital Health's publication criteria as evidenced by the sound methodology, ethically rigorous research with realistic conclusion in study: - see lines 57 -565) 2. Yes, supported by the methodology (lines 226 - 429) 3. Yes, the authors made all data underlying the findings in their manuscript as per statement at the start of the manuscript file. 4. Yes, the manuscript presented in standard English- readable, great syntax, clear and organized

We would like to sincerely thank dr. Audrey Blackwood for the positive assessment of our manuscript.

**REVIEWER 3:**

We would like to sincerely thank Towhida Ahsan for the suggestions to improve our manuscript. Our responses to the comments can be found below.

**COMMENT 1: I read the study with interest and found it to be valuable. However, I noticed that the authors did not provide a clear explanation of the generalization procedure, which should be addressed.**

Amended. The aim of our research was not to generate population estimates and we did not employ a sampling strategy that would allow us to do so. Given these limitations, we did not employ a formal generalization procedure. Nevertheless, we now more elaborately discuss (also in line with other reviewers’ comments) in our discussion section the degree to which our findings may or may not be generalized to CT under different circumstances and the overall population of (Dutch) PHPs (L537 - 565):

*‘At the same time, we are aware that the results from this study are likely strongly influenced by the circumstances under which CT was performed at the time that this study was conducted. For example, most PHS in the Netherlands had insufficient human resources available (considering the number of daily confirmed new cases) to thoroughly conduct CT. As such, making the CT-process more feasible and efficient was an important task for PHS, which we believe to be reflected in the fact that related items (speed, workload, difficulty) ranked relatively highly in the VIR of all DCTS-tools. It may well be that the prioritization of items will be different under different circumstances, both in terms of the stage of an outbreak (e.g., a new or an established outbreak) and in terms of the specific disease at hand. However, considering the similarities in results between this study, previous exploratory research that we conducted before the COVID-19 pandemic on the same topic, and several other studies (as previously outlined), we still strongly believe that our results and recommendations are relevant regarding the application of DCTS-tools beyond the COVID-19 pandemic.*

*Since we mainly relied on referrals and snowball sampling to distribute the online questionnaire, we were unable to calculate a response rate. In addition, we have no precise insights into characteristics of the overall population of PHPs at the time this study was conducted. This means that we cannot accurately estimate to what degree our study may have suffered from selection bias. Furthermore, our sample consisted mainly of temporary contact tracers, which may not represent the overall population of PHPs under ‘normal’ (i.e., non-pandemic) circumstances. This should be kept in mind when interpreting this study’s results and findings.*

*Nevertheless, we would like to emphasize that we managed to collect data from a large number of PHPs (N=641), representing all relevant socio-demographic and professional backgrounds as described in Table 1. Even though our sample consisted largely of temporary contact tracers, our RF-models show that PHPs’ socio-demographic and professional characteristics had a negligible effect on their intention to use DCTS-tools. This indicates that potential bias in our study related to over- or underrepresentation of professional backgrounds may be limited. As previously described, we also established that the potential bias in our study related to respondent dropout is limited. Therefore, we argue that we have obtained a good overall picture of PHPs’ attitudes and the factors influencing their intention to use DCTS-tools in the Netherlands, especially under pandemic circumstances and/or when large-scale CT is required.’*

In addition, in the ‘Comparison of findings with Literature’ section we compare our findings to several international studies conducted in a range of different settings. We conclude this section by outlining that PHPs’ perspectives and needs regarding the use of DCTS-tools may be similar in different countries and settings (L492 - 497):

*‘The similarities between these studies and the results from our study indicate that PHPs’ may have similar perspectives and needs regarding the use of DCTS-tools across different countries and in the context of different communicable diseases. For example, (personal) support for cases and contacts, and considering their preferences, willingness, and skills in the application of digital CT-tools is a recurring theme that consistently seems to lead PHPs to consider DCTS-tools as an addition to - rather than a replacement of - the ‘traditional’ CT-approach.’*

**COMMENT 2: The study was conducted between February and April 2022, during a non-pandemic situation. However, the authors did not mention why they chose online questionnaires instead of face-to-face interviews.**

Clarified: We apologize for any confusion in this regard. The study was performed during a pandemic situation, or at least at a time at which contact tracing for COVID-19 was still performed at a very large scale in the Netherlands (although in a scaled down fashion). We now more clearly describe this in the methods section ‘Setting and study population’ (L150 - 159):

*‘This study was performed between February and April 2022. At that time, the number of newly identified COVID-19 cases per day was particularly high in the Netherlands, but regulations concerning contact restrictions to reduce transmission of COVID-19 (e.g., social distancing, maximum group sizes) were gradually being lifted due to increasing vaccination rates, diminishing hospitalizations rates of COVID-19 patients and the emergence of less severe variants of the virus. CT was still implemented on a large scale, but mostly in a (very) scaled-down manner. Therefore, PHS had temporarily hired many new contact tracers (e.g., medical students) and focused the responsibilities of nurses and physicians towards relatively complex clusters and the overall coordination of CT. Although national guidelines for CT were in place, the precise execution of CT (e.g., the degree to which CT was scaled down) often differed between PHS, depending on regional caseload and available personnel for CT.’*

Regarding our choice for online questionnaires instead of interviews: we already conducted interviews with key-informants in a previous (separate) qualitative study. The aim of this study was to investigate our previous qualitative findings in a larger population of PHPs, so to establish and quantify determinants of PHPs’ intention to use DCTS-tools in practice. To this purpose, we believe that online questionnaires are an appropriate and sufficient method.

**COMMENT 3: Data were collected from the PHPs involved in CT for COVID-19, which could have resulted in recall bias. Unfortunately, the authors did not address this issue in the limitations section.**

Clarified: see also our reaction regarding comment 2. Since we collected data from PHPs who were directly involved in CT for COVID-19 at the time that this study was performed, we believe that recall bias is less of an issue in this study.

**COMMENT 4. The study included a large number of temporary contact tracers (538 or 83.9%) and some participants involved as young as 16 years old. Thus, the sample group may not accurately reflect actual public health professionals.**

Amended. We agree that our study included a large number of temporary contact tracers, which may have impacted our study findings. However, this may mostly be true when drawing conclusions with regards to CT under ‘normal’ (i.e., non-pandemic) circumstances. We believe that our sample may reflect the population of PHPs under pandemic circumstances quite well, especially when large scale CT is required that exceeds the human resource capacity of PHS. In addition, our random forest analyses suggest that the professional background of respondents had a negligible effect in determining PHPs’ intention to use DCTS-tools. This further indicates that our results may reflect the overall population of PHPs sufficiently well, especially under pandemic circumstances. We now more elaborately discuss this in the limitations section (L549 - 565):

*‘Since we mainly relied on referrals and snowball sampling to distribute the online questionnaire, we were unable to calculate a response rate. In addition, we have no precise insights into characteristics of the overall population of PHPs at the time this study was conducted. This means that we cannot accurately estimate to what degree our study may have suffered from selection bias. Furthermore, our sample consisted mainly of temporary contact tracers, which may not represent the overall population of PHPs under ‘normal’ (i.e., non-pandemic) circumstances. This should be kept in mind when interpreting this study’s results and findings.*

*Nevertheless, we would like to emphasize that we managed to collect data from a large number of PHPs (N=641), representing all relevant socio-demographic and professional backgrounds as described in Table 1. Even though our sample consisted largely of temporary contact tracers, our RF-models show that PHPs’ socio-demographic and professional characteristics had a negligible effect on their intention to use DCTS-tools. This indicates that potential bias in our study related to over- or underrepresentation of professional backgrounds may be limited. As previously described, we also established that the potential bias in our study related to respondent dropout is limited. Therefore, we argue that we have obtained a good overall picture of PHPs’ attitudes and the factors influencing their intention to use DCTS-tools in the Netherlands, especially under pandemic circumstances and/or when large-scale CT is required.’*

Regarding the age of respondents: we made a mistake and apologize for any confusion in this regard. The minimal age to participate in this study (and the youngest participants) actually were 18 years old. We accidentally wrote 16 in the manuscript, since this is the standard minimal age at which individuals in the Netherlands are legally allowed to participate in research without requiring additional informed consent procedures. We corrected this in the manuscript.

Nevertheless, although some individuals were indeed young, we would like to point out that the average age of participants was 40.7 and that 50% of our respondents were aged between 28 and 53 years old. In addition, age had no meaningful impact on PHPs’ intention (as evidenced by our random forest plots). As such, we do not believe that respondents’ age was a troublesome factor in our study.

In summary, although we do agree with Towhida Ahsan that our sample may not represent the overall population of PHPs under non-pandemic circumstances in terms of their professional background, we believe that our results still provide a good overall picture of (the drivers of) PHPs’ intention to use DCTS-tools., especially under pandemic circumstances or when very large-scale CT is required.

**COMMENT 5: To make the study more reliable, a number of Key Informant Interviews should have been conducted.**

Clarified. See also our response to comment 2. We did not conduct key-informant interviews in this study since this study was already based on findings resulting from key-informant interviews (find article [here](https://bmchealthservres.biomedcentral.com/articles/10.1186/s12913-022-08764-y)). In addition, our results and findings are strongly in line with our previous qualitative findings. We, therefore, believe that additional interviews would be redundant and not in line with the aims of our study.
