# Supplementary material for: Determinants of Dutch public health professionals’ intention to use digital contact tracing support tools: A cross-sectional online questionnaire study
Source: PLOS Digit Health. 2024 Feb 14;3(2):e0000425. doi: 10.1371/journal.pdig.0000425 (PMC10866487; doi:10.1371/journal.pdig.0000425)
Supplement: Attachment — Submitted filename: Response to Reviewers_V2_28112023.docx [file pdig.0000425.s006.docx]

National Institute for Public Health and the Environment

Centre for Infectious Disease Control

National Coordination Centre for Communicable Disease Control

Antonie van Leeuwenhoeklaan 9

3720 BA Bilthoven

The Netherlands

[yannick.helms@rivm.nl](mailto:yannick.helms@rivm.nl)

28^th^ of November 2023

Dear Haleh Ayatollahi,

We are very pleased to hear that the Reviewers are satisfied with our revised manuscript titled: ‘Determinants of Dutch public health professionals’ intention to use digital contact tracing support tools: a cross-sectional online questionnaire’. Based on the additional minor comments to improve our manuscript, we made several small adjustments. Our responses to the comments are in blue:

1. **Please follow the journal instructions for preparing the manuscript, e.g. Unstructured/structured abstract and appropriate headings/subheadings:**

Amended. We changed the format of the abstract and the headings/subheadings in accordance with the PLOS Digital Health guidelines and online examples.

2. **Please add appropriate keywords using the MeSH-terms.**

Amended. In the online submission system, we now include only appropriate MeSH-terms.

3. **Please add a reference to the 1st paragraph of the introduction.**

Amended. We included two references at the end of the first paragraph and updated the reference list (reference numbers 1 and 2). In addition, we checked the reference list and changed the formatting of several references to comply with the PLOS Digital Health guidelines. One reference that was previously only available on *PsyArXiv* was published in *Health Psychology and Behavioral Medicine* during the review-process (reference number 27). We updated this reference accordingly.

4. **Please ensure that the aim of the study has been clearly mentioned in the abstract and introduction.**

Amended. The aim of our study is now explicitly stated in the abstract (end of first paragraph) and in the introduction (last paragraph).

We hope that our revised manuscript now fully complies with all requirements of PLOS Digital Health. We sincerely appreciate your time and efforts. All authors have seen and approved the revised manuscript.

Yours faithfully,

Yannick Helms, M.Sc.

(Corresponding author)
